# Novel probiotic preparation with *in vivo* gluten-degrading activity and potential modulatory effects on the gut microbiota

**DOI:** 10.1128/spectrum.03524-23

**Published:** 2024-06-11

**Authors:** Olga Nikoloudaki, Giuseppe Celano, Andrea Polo, Claudia Cappello, Lena Granehäll, Alice Costantini, Mirco Vacca, Bodo Speckmann, Raffaella Di Cagno, Ruggiero Francavilla, Maria De Angelis, Marco Gobbetti

**Affiliations:** 1 Faculty of Agricultural, Environmental and Food Sciences, Free University of Bozen-Bolzano, Bolzano, Italy; 2 Department of Soil, Plant and Food Sciences, University of Bari Aldo Moro, Bari, Italy; 3 Evonik Operations GmbH, Creavis, Essen, Germany; 4 Interdisciplinary Department of Medicine-Pediatric Section, University of Bari Aldo Moro, Ospedale Pediatrico Giovanni XXIII, Bari, Italy; National Institutes of Health, Bethesda, Maryland, USA

**Keywords:** gluten, celiac disease, gluten-free diet (GFD), probiotic, digestion, lactobacilli, *Bacillus* sp., residual gluten, qPCR, fecal metabolome

## Abstract

Gluten possesses unique properties that render it only partially digestible. Consequently, it exerts detrimental effects on a part of the worldwide population who are afflicted with celiac disease (1%) or related disorders (5%), particularly due to the potential for cross-contamination even when adhering to a gluten-free diet (GFD). Finding solutions to break down gluten during digestion has a high nutritional and social impact. Here, a randomized double-blind placebo-controlled *in vivo* challenge investigated the gluten-degrading activity of a novel probiotic preparation comprising lactobacilli and their cytoplasmic extracts, *Bacillus* sp., and bacterial protease. In our clinical trial, we collected feces from 70 healthy volunteers at specific time intervals. Probiotic/placebo administration lasted 32 days, followed by 10 days of wash-out. After preliminary GFD to eliminate residual gluten from feces, increasing amounts of gluten (50 mg–10 g) were administered, each one for 4 consecutive days. Compared to placebo, the feces of volunteers fed with probiotics showed much lower amounts of residual gluten, mainly with increased intakes. Probiotics also regulate the intestinal microbial communities, improving the abundance of genera pivotal to maintaining homeostasis. Quantitative PCR confirmed that all probiotics persisted during the intervention, some also during wash-out. Probiotics promoted a fecal metabolome with potential immunomodulating activity, mainly related to derivatives of branched-chain amino acids and short-chain fatty acids.

**IMPORTANCE:**

The untapped potential of gluten-degrading bacteria and their application in addressing the recognized limitations of gluten-related disorder management and the ongoing risk of cross-contamination even when people follow a gluten-free diet (GFD) emphasizes the significance of the work. Because gluten, a common protein found in many cereals, must be strictly avoided to stop autoimmune reactions and related health problems, celiac disease and gluten sensitivity present difficult hurdles. However, because of the hidden presence of gluten in many food products and the constant danger of cross-contamination during food preparation and processing, total avoidance is frequently challenging. Our study presents a novel probiotic preparation suitable for people suffering from gluten-related disorders during GFD and for healthy individuals because it enhances gluten digestion and promotes gut microbiota functionality.

## INTRODUCTION

The main storage proteins of wheat grains, glutenins, and gliadins form gluten upon the addition of water, which determines the rheological behavior of the dough (1). Hydrated gliadins impact dough extensibility and viscosity, while hydrated glutenins determine dough cohesiveness and elasticity (2). Gluten tolerates heating and acts as a binding and extending agent, which makes it also suitable as a texture, flavor, and moisture retention ingredient (3).

Because of these technological properties and its ubiquitous nature, gluten intake spreads worldwide, being the major food protein consumed in Western diets (up to 20 g gluten/day) (4). But gluten also has unique and unusual features. It resists complete luminal digestion by gastric, pancreatic, and intestinal brush border enzymes and is susceptible to post-translational modification (deamidation) by mucosal transglutaminases. Apart from partial digestion, gluten *per se* has a negative impact on a part of the worldwide population, which mainly manifests in the form of celiac disease (CD) (1%) (5) and other gluten-related disorders (up to 5%) (6). Sensitivity to gluten can occur at any time after its dietary introduction or any age later in life (7). While a gluten-free diet (GFD) is the sole therapeutic option for individuals affected by CD and is commonly recommended for other gluten-related disorders, it is important to clarify that the primary motivation for adhering to a GFD is the necessity to manage CD symptoms. Despite the imperative nature of the diet for those with CD, complete avoidance of gluten-containing foods remains challenging, if not unattainable, due to factors such as food cross-contamination, insufficient food labeling, social constraints, and economic and distribution challenges (8). Even gluten-free products might contain traces of gluten exceeding the safe intake threshold (<20 ppm). It is estimated that not more than 45%–90% of CD patients effectively adhere to the GFD (9), with daily consumption of gluten deriving solely from cross-contamination of approximately 5–50 mg (10).

Once liberated during digestion, gluten peptides with a size bigger than 10 amino acids might act as immunogenic (11). Glutenins and mainly gliadins contain at least 50 different crypted immunogenic peptides, which are resistant to further hydrolysis (1). This is mainly owing to an unusually high content of proline residues, which, because of the cyclic structure, hydrogen-free N-terminus, and side *cis* chains (12, 13), form peptide bonds resistant to all mammalian gastrointestinal peptidases (12, 14). Overall, the incomplete gluten hydrolysis slows down the digestion of healthy people (14). More specifically, it leads to the formation of epitopes triggering food disorders in predisposed people (8). Theoretically, up to nine peptidases are necessary to hydrolyze all gluten oligopeptides where proline is potentially located at different positions. Humans do not possess this enzyme portfolio (8). Previously (8), we comprehensively screened hundreds of bacterial strains for their ability to degrade gluten under simulated gastrointestinal conditions. Then, we selected a probiotic preparation comprising lactic acid bacteria, bacilli, bacterial cytoplasmatic extracts, and bacterial proteases, highly efficient in degrading gluten *in vitro*.

There is no doubt that the human gut microbiota assists gluten metabolism (15). The duodenum receives food proteins and peptides, whose preliminary digestion starts at the stomach level. These derivatives, including refractory gluten peptides, are likely to provide nitrogen nourishment to resident bacteria in the small intestine (1, 16). Some gut colonizers (e.g., *Neisseria flavescens* and *Pseudomonas aeruginosa*) led to increased contents of gluten epitopes (8). On the contrary, *Lactobacillus* spp., which were previously isolated from the duodenum of healthy individuals, degraded gluten peptides in mice and decreased their immunogenicity (17). Even if the mechanism for gluten detoxification by probiotics has yet to be unraveled completely, the literature shows promising *in vitro* and *ex vivo* results (18). Administration of probiotic bifidobacteria and lactobacilli protected intestinal cells from gliadin toxicity by preventing the increase of the epithelial permeability and stimulating regulatory T cells to synthesize IL-10, which promoted intestinal gluten metabolism (19). Selected probiotic lactobacilli with complementary peptidase activities *in vitro* hydrolyzed gluten immunogenic peptides (20). Baked goods made from hydrolyzed wheat flour and manufactured with selected sourdough lactobacilli and fungal proteases were absolutely safe for CD patients (21).

Here, we describe a randomized, placebo-controlled double-blind 42-day *in vivo* challenge on 70 healthy volunteers following the Mediterranean diet. Feeding volunteers with increasing amounts of gluten, we confirmed for the first time the gluten-degrading activity of our novel probiotic preparation in humans. Moreover, our probiotic preparation altered the gut microbiota, boosting the diversity of genera essential for preserving homeostasis. This was also reflected in the gut microbiota functionality, which may favor immunomodulatory responses. Our gluten-targeted probiotic preparation may benefit both people with gluten-related diseases even under GFD and healthy people by enhancing gluten digestion.

## RESULTS

### Recruitment and sampling size calculation

**Fig 1:**
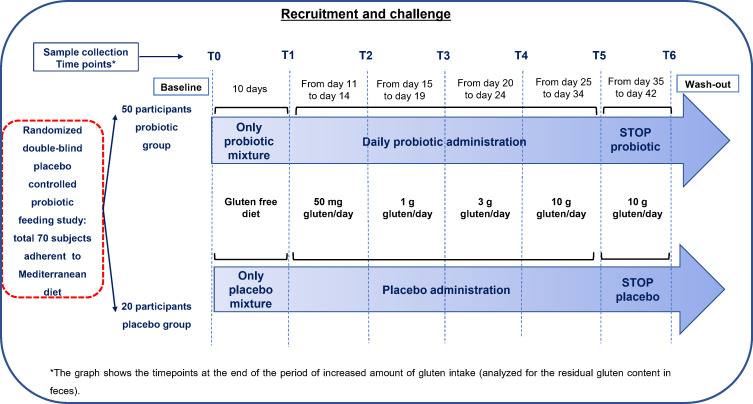
Recruitment and study design of the *in vivo* challenge. Description of participant division per group, administration of probiotic–placebo, and gluten intake throughout the duration of the study.

Recruitment lasted from October 2020 to May 2021 because of the COVID-19 emergency. The study was a randomized, double-blind, placebo-controlled *in vivo* challenge where increasing amounts of gluten were administrated to volunteers following GFD (Fig. 1; Fig. S1: CONSORT flow diagram). Considering the inherent difficulty of recruiting healthy participants willing to undergo GFD and maintain this diet with fixed amounts of gluten intake for 42 days, we decided to have unequal sampling sizes for the probiotic and placebo groups (22). Due to the lack of any pilot data, the target sample size of 40–70 volunteers was decided based on literature studies, where the effectiveness of probiotic administration was assessed (23, 24). The final number of eligible participants was 70. As estimated by package “pwr” (R version 4.1), this number was sufficient to show an effect size >0.70 and to compare the probiotic (*n* = 50) and placebo (*n* = 20) groups at significance level α = 0.05 and 80% power. Randomization lists were computer-generated by a statistician and given to researchers. The randomization took place in a way that researchers, doctors, and volunteers were blinded. Apart from capsules and instructions, volunteers also received a food questionnaire to record dietary habits before the intervention. The Mediterranean Dietary Serving Score (MDSS, ranging from 0 to 24) was used considering the latest update of the Mediterranean Diet Pyramid (25, 26). MDSS evidenced a significant discriminative capacity between adherents and non-adherents (optimal cut-off point = 13.50; sensitivity = 74%; specificity = 48%). The resulting mean for the 70 volunteers was 15.45, which demonstrated a satisfactory adherence to the Mediterranean diet.

### Probiotics degrade gluten during digestion

**Fig 2:**
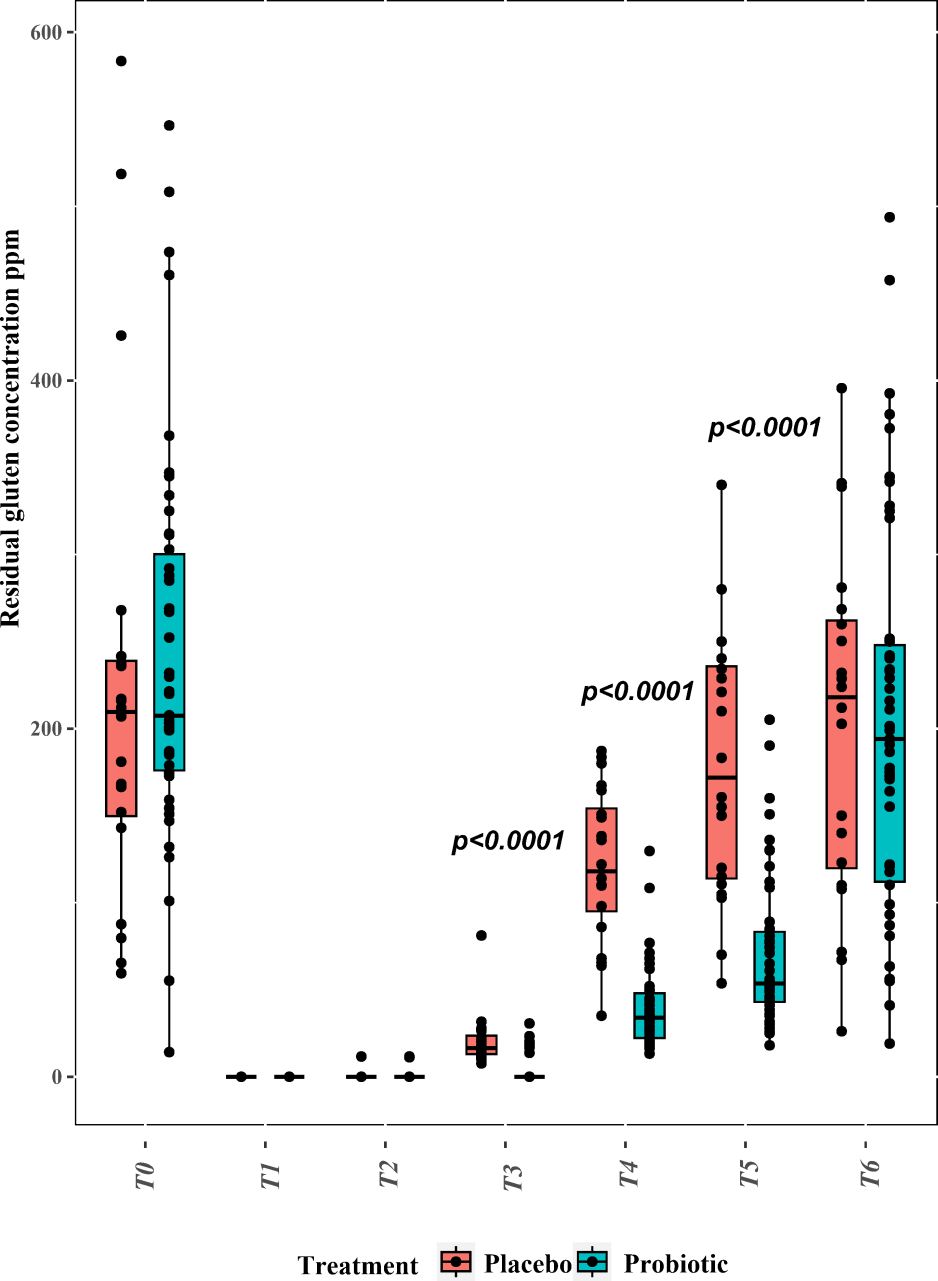
Average gluten concentrations (ppm) in feces of volunteers belonging to probiotic (*n* = 50; orange color bar) and placebo (*n* = 20; blue color bar) groups. Sampling points at the *x*-axis correspond to baseline (**T0**), 10 days of GFD (**T1**), 4 days of 50 mg/day gluten intake (**T2**), 4 days of 1 g/day gluten intake (**T3**), 4 days of 3 g/day gluten intake (**T4**), and 20 days of 10 g/day gluten intake (**T5**) of which the 10 last days were the wash-out (**T6**). Differences between the groups for each time point were estimated by the non-parametric Mann–Whitney test (exact *P* < 0.0001). The data are the means of two independent analyses ± standard deviations (*n* = 2) per sample.

We investigated the gluten-degrading activity of our probiotic preparation by quantifying residual gluten in feces from the probiotic (*n* = 50) and placebo (*n* = 20) groups. The competitive ELISA from Ridascreen was used. Sampling points were from T0 to T6, where T2 to T6 corresponded to intakes of increasing gluten amounts. At the start (T0), the feces of volunteers from the probiotic and placebo groups had an almost similar content of gluten: 248.93 ± 9.42 vs 223.32 ± 14.01 ppm (Fig. 2). No gluten was detected at T1, reinforcing that all volunteers adhered to GFD. After 4 days of 0.05 g/day gluten intake (T2), only the feces of two volunteers belonging to the probiotic group had detectable amounts of gluten (average group value of 0.46 ± 0.24 ppm). For the placebo group, only one volunteer was positive for gluten (11.62 ± 0.40 ppm). After 4 days of 1 g/day gluten intake (T3), only seven volunteers belonging to the probiotic group showed detectable amounts of gluten in the feces (average group value of 2.803 ± 4.38 ppm). On the contrary, all feces of all volunteers from the placebo group showed residual gluten (average group value of 20.89 ± 3.98 ppm), and the difference between the average value of gluten detected was statistically different (*P* < 0.0001). The difference between the two groups persisted (*P* < 0.0001) when the daily gluten intake increased to 3 g/day and then to 10 g/day. At T4 and T5, the gluten content from the feces of the placebo group increased to average values of 122.13 ± 5.14 ppm and 179.02 ± 17.92 ppm, respectively. Much lower was the content of gluten found in the feces from the probiotic group: 38.82 ± 3.80 ppm and 69.51 ± 5.56 ppm, respectively. After wash-out (T6) without probiotic or placebo administration, the content of gluten did not show statistical differences between the two groups: 199.68 ± 11.28 ppm vs 201.54 ± 18.56 (probiotic vs placebo).

### Volunteers sub-setting

Excluding T0, T1, and T2, cumulative data (*n* = 70) were used to determine the lower or 25th empirical quartile (Q1), median of each data set (Q2), and upper or 75th empirical quartile (Q3). T0 was excluded because volunteers followed their own diet; T1 was removed because all volunteers adhered to GFD and had almost no residual gluten in their feces, likewise for T2. For the probiotic and placebo groups, quartiles were as follows: Q1 = 33.5 vs 32.46 ppm, Q2 = 55 vs 114.51 ppm, and Q3 = 133.19 vs 208.18 ppm. Randomly, 29 out of 50 and 14 out of 20 volunteers belonging to the probiotic and placebo groups were selected based on the range of each quartile.

### Probiotics remain active during administration and alter the microbial richness

**Fig 3:**
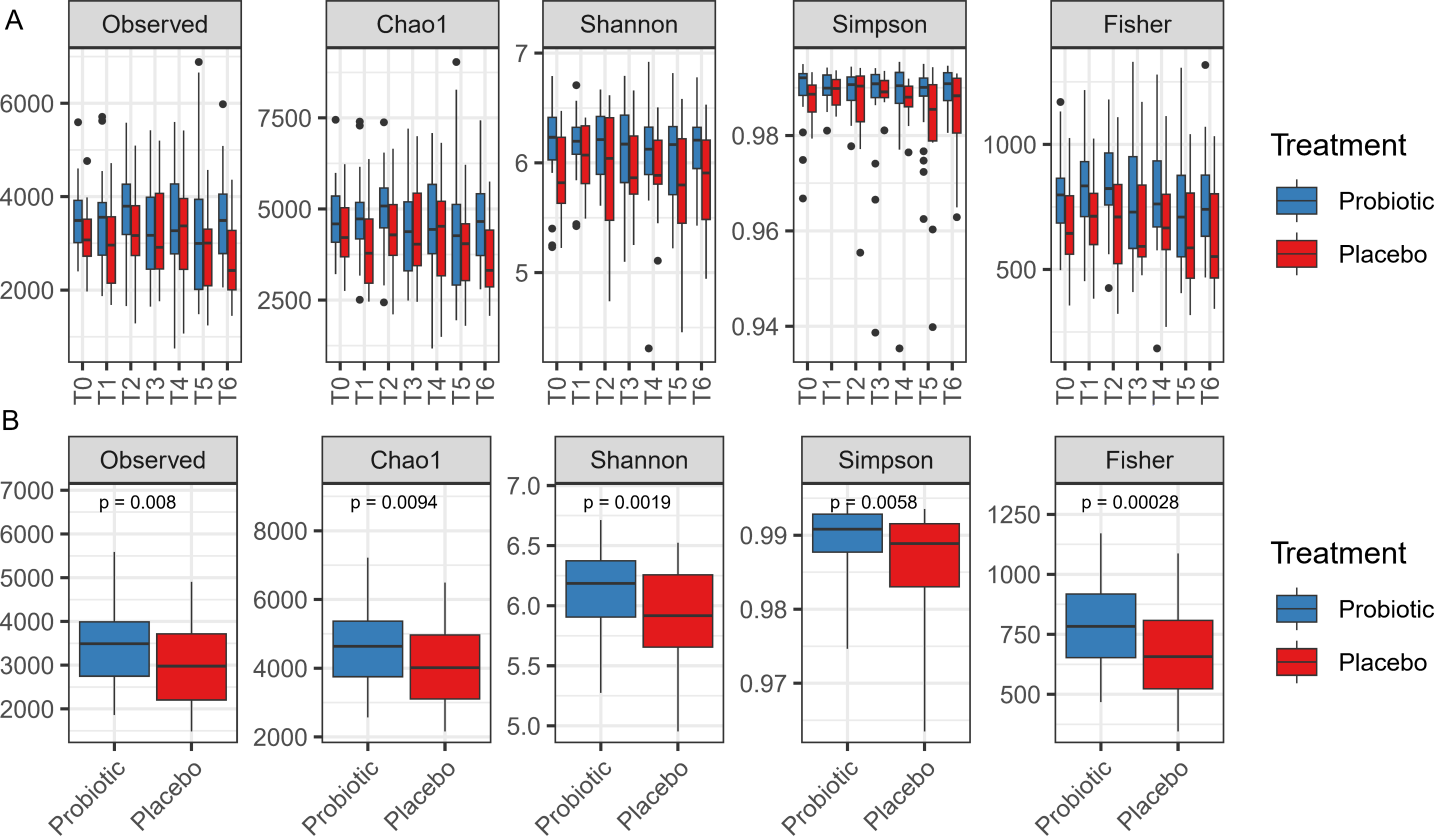
Alpha diversity metrics of probiotic and placebo groups. Pairwise Wilcoxon signed-rank test was used to compare means of placebo and probiotic groups (*P* < 0.05). (**A**) Observed operational taxonomic unit (OTU) counts and richness measures (Chao1, Shannon, Simpson, and Fisher) between volunteers, who received probiotic or placebo treatment, divided by time points: baseline (**T0**), 10 days of GFD (**T1**), 4 days of 50 mg/day gluten intake (**T2**), 4 days of 1 g/day gluten intake (**T3**), 4 days of 3 g/day gluten intake (**T4**), and 20 days of 10 g/day gluten intake (**T5**), of which the last 10 days were the wash-out (**T6**). **(B**) Alpha diversity indexes and respective significance values of merged termporal results per treatment group exluding T0.

The gut microbiota is one of the most investigated components of the gastrointestinal tract and was demonstrated to be a key driver during the development of intestinal inflammation. Therefore, we investigated whether our probiotic preparation could regulate the microbial richness of the gut intestinal microbiota. Using the sub-set of volunteers, we obtained 18,513,963 sequence reads from bacterial 16S rRNA gene V3–V4 amplicons, with an average of 89,009 ± 51,327 reads per feces. After filtering, 11 phyla, 58 families, 147 genera, and 216 species were identified. The most abundant families of both probiotic and placebo groups were *Lachnospiraceae* (average 45.1% and 50.9%, respectively), *Ruminococcaceae* (average 17.3% and 14.9%), and *Bifidobacteriaceae* (4.11% and 4.50%). The topmost abundant genera found in volunteers fed with probiotics were *Blautia* (10.8%), *Faecalibacterium* (8.87%), and *Bifidobacterium*. For the placebo group, the top genera were *Blautia* (15.3%), *Faecalibacterium* (7.84%), and *Agathobacter* (5.75%) (Fig. S2). Longitudinal samples, ranging from T0 (baseline) to T6, were analyzed and compared between volunteers in both the probiotic and placebo groups. No significant differences were found between samples at baseline (T0) and also for the rest of the time points (Fig. 3A). When time points were grouped together (T1–T6), compared to placebo, probiotics promoted higher species count and richness (pairwise Wilcoxon rank-sum test, *P* < 0.05).

**Fig 4:**
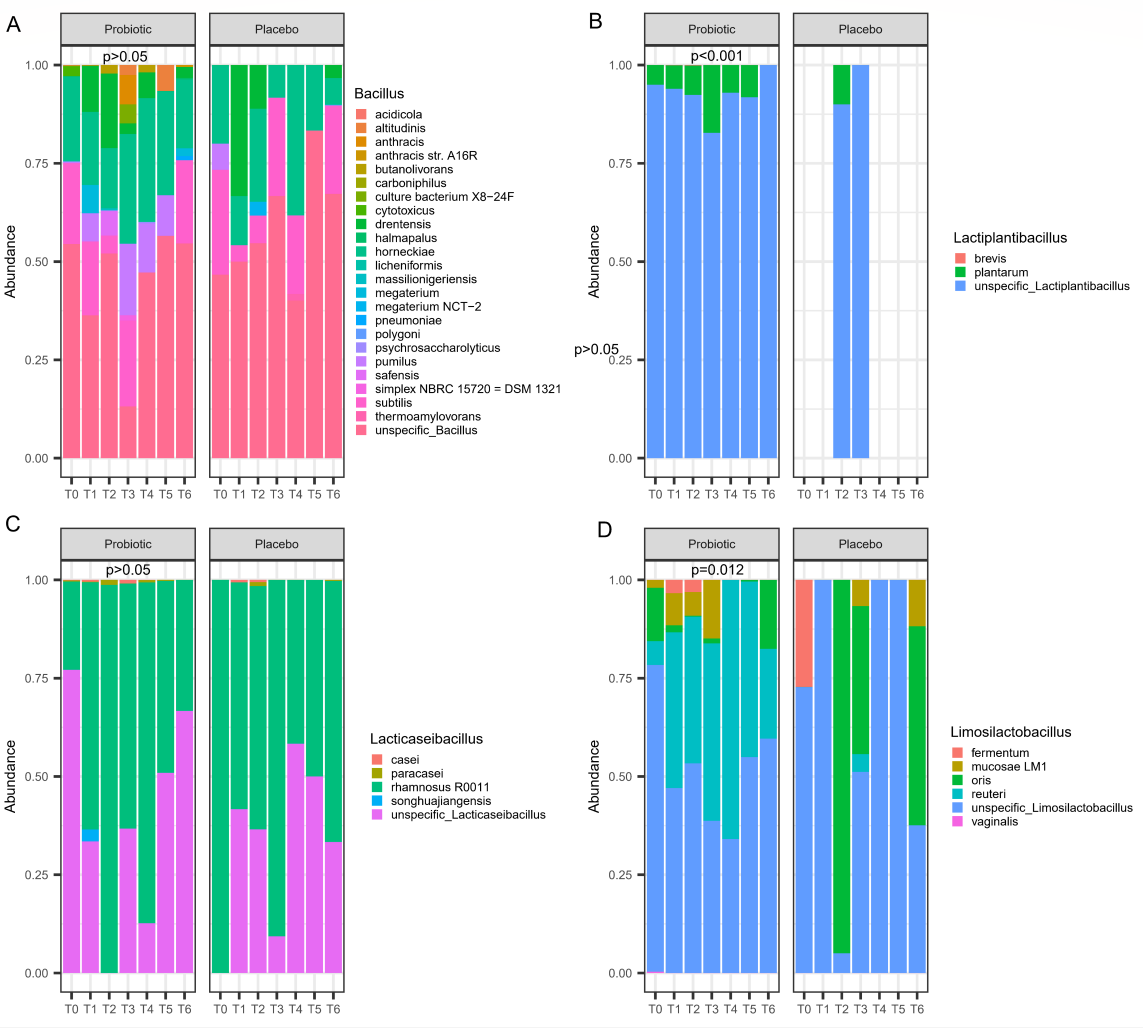
Differences in species abundances of the four genera included in the administered probiotic preparation. Time points analyzed were as follows: baseline (**T0**), 10 days of GFD (**T1**), 4 days of 50 mg/day gluten intake (**T2**), 4 days of 1 g/day gluten intake (**T3**), 4 days of 3 g/day gluten intake (**T4**), and 20 days of 10 g/day gluten intake (**T5**), of which the last 10 days were the wash-out (**T6**). Panels: (A) *Bacillus*, (**B**) *Lactiplantibacillus*, (**C**) *Lacticaseibacillus*, and (D) *Limosilactobacillus*. Pairwise Wilcoxon signed-rank test was used to compare means of placebo and probiotic groups (*P* < 0.05). To validate the presence of probiotic species in fecal samples, we conducted BLAST analysis on OTUs assigned to Lactobacillaceae and Bacillaceae families, ensuring >99% sequence similarity with the reference genomes of the probiotic species.

**Fig 5:**
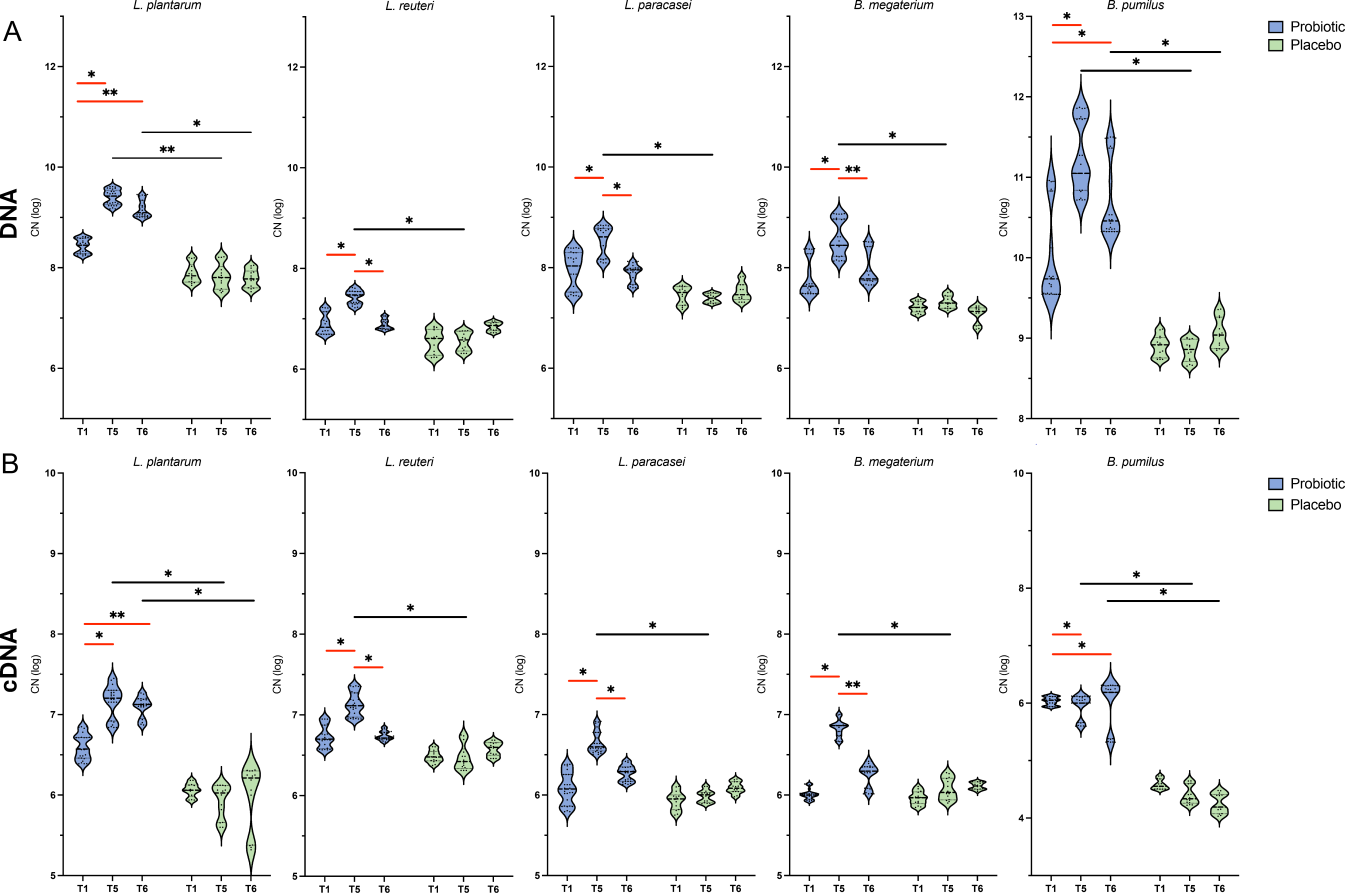
Persistence of probiotic preparation. Copy number (CN log) obtained from qPCR analysis carried out on (A) total DNA and (**B**) cDNA extracted from fecal material of healthy volunteers allocated in the probiotic or placebo group. In the probiotic group, significant differences (*P*-values <0.05; two-tailed Student’s *t*-test) between different time points [10 days of GFD (**T1**) and 20 days of 10 g/day gluten intake (**T5**), of which the last 10 days were the wash-out (**T6**)] are showed with red lines. Based on the comparison of the same time point between the probiotic and placebo groups, significant differences (^*^P-values <0.05; ^**^P-values <0.01, two-tailed Student’s *t*-test) are showed with black lines.

The principal coordinate analysis (PCoA) using Bray–Curtis dissimilarity did not allow for discerning differences in community diversity between the probiotic and placebo groups. However, permutational multivariate analysis of variance analysis (PERMANOVA) analysis showed that microbial communities slightly differ between (*P* = 0.001) treatments (probiotic vs placebo) and within each treatment (probiotic: *P* = 0.011; placebo: *P* = 0.043) according to time points. Thirty-eight genera were found by linear discriminant analysis effect size (LEfSe) to be differentially abundant between the two groups (Fig. S3; Table S1). The majority of differentially abundant genera (87%) were found in volunteers fed with probiotics, including *Coprococcus*, *Streptococcus,* and *Lactococcus*. Only five genera were highly abundant in the placebo group, mainly *Blautia*, *Oscillibacter,* and *Flavonifractor*. Assessing the differentially abundant genera over time and per group (probiotic vs placebo), no statistically significant differences were found. DNA metabarcoding analysis showed the presence of all genera from the probiotic preparation during the intervention and subsequent wash-out. Three of the four genera, *Bacillus*, *Lactiplantibacillus,* and *Limosilactobacillus*, were all more abundant in the feces of volunteers fed with probiotics (Fig. 4A through D). The differences were statistically significant for *Lactiplantibacillus* (*P* < 0.001) and *Limosilactobacillus* (*P* = 0.012). Figure 5 gives an overview of the quantitative results obtained by species-specific quantitative PCR (qPCR) targeting solely probiotic species. The primers selected (27–29), along with their target regions and amplification conditions, are detailed in Table 1. At T1, the quantities [log copy numbers (CNs)] did not differ between the probiotic and placebo groups (Fig. 5A). Significant differences (*P* < 0.05) between groups were detected at the end of the intervention (T5). Compared to T1, CN increased for all probiotic species. At T6, the values of CN tended to significantly decrease (*P* < 0.05) for the probiotic group. *Limosilactobacillus reuteri*, *Lacticaseibacillus paracasei*, and *Bacillus magaterium* did not exhibit differences between T1 and T6. At T6, *Lactiplantibacillus plantarum* and *Bacillus pumilus* continued to be present in the feces of volunteers fed with probiotics. The persistence of the probiotic species was confirmed using the complementary DNA (cDNA) template (Fig. 5B). At T1, the qPCR analysis confirmed the lack of differences between the probiotic and placebo groups. The cell viability of all amplified probiotics was higher at T5 than T1 for all feces of volunteers fed with the probiotic preparation. On the contrary, no differences were found for the placebo group when T5 was compared to T1. By comparing T5 and T6 within the probiotic group, a significant (*P* < 0.05) decrease of CN was observed for *Ls. reuteri*, *Lc. paracasei*, and *B. magaterium*. On the contrary, *Lp. plantarum* and *B. pumilus* maintained similar viabilities in the feces. For the placebo group, the values of CN did not significantly vary throughout the challenge.

**TABLE 1:** List of species-specific primers for monitoring the probiotic preparation with quantitative real-time PCR and related working features

Species	Primers	Primer sequence (5′–3′)	Target gene	Product size (bp)	T annealing (°C)	Reference
*Lp. plantarum*	Lp-F	AAAATCATGCGTGCGGGTAC	*pyrG*	261	55	(27)
	Lp-R	ATGTTGCGTTGGCTTCGTCT				
*Ls. reuteri*	Lreu-1	CAGACAATCTTTGATTGTTTAG	*16S-23S spacer region*	305	60	(27)
	Lreu-4	GCTTGTTGGTTTGGGCTCTTC				
*Lc. paracasei*	PC2a	GGATTGGGTTTTGCGTGATGGTCGC	*mutL*	261	68	(27)
	CPRrev	TGCATTTCCCCGCTTTCATGACT				
*B. megaterium*	BmphaC015F	CGTGCAAGAGTGGGAAAAAT	*phaC*	900	64	(28)
	BmphaC931R	TCGCAATATGATCACGGCTA				
*B. pumilus*	rpoB_F	ATCGAAACGCCTGAAGGTCCAAACAT	*rpoB*	1,200	52	(29)
	rpoB_R	ACACCCTTGTTACCGTGACGACC				

### The fecal metabolome of volunteers fed with probiotics: potential immunomodulating compounds

**Fig 6:**
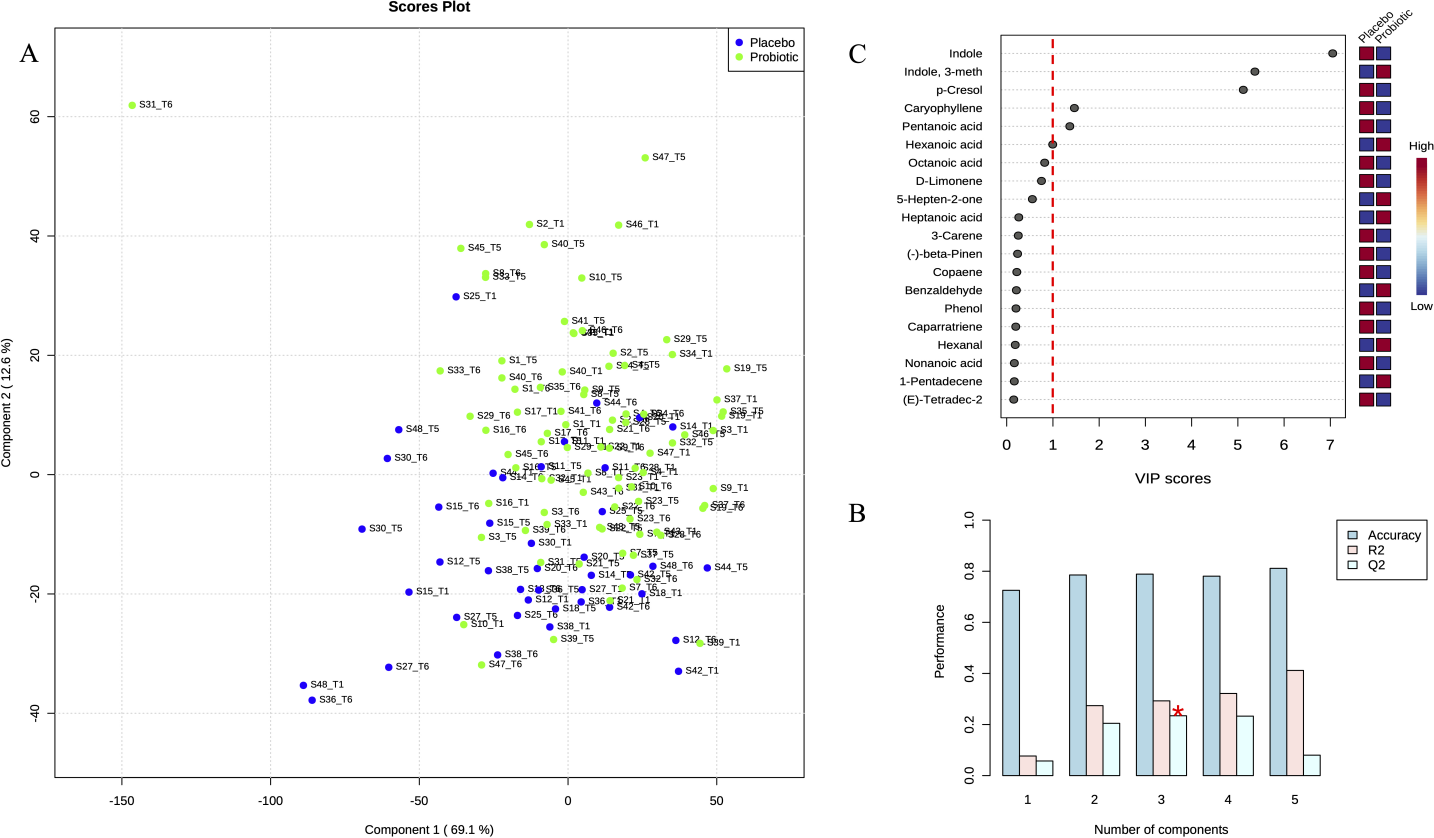
PLS-DA based on VOC abundance normalized matrix. (**A**) First and second components were used to plot samples onto a bidimensional graph. Green and blue colors indicate probiotic (*n* = 29) and placebo (*n* = 14) groups, respectively. (**B**) Cross-validated accuracy/R2/Q2 coefficients produced as a result of a permutation analysis between five predictive components. (**C**) PLS-DA VIP plot was computed based on the complete panel of detected VOC. Mostly important metabolite features identified by PLS-DA are ranked at the top. Blue and red boxes on the right indicate relative concentration of corresponding VOC for samples belonging to placebo and probiotic groups.

**Fig 7:**
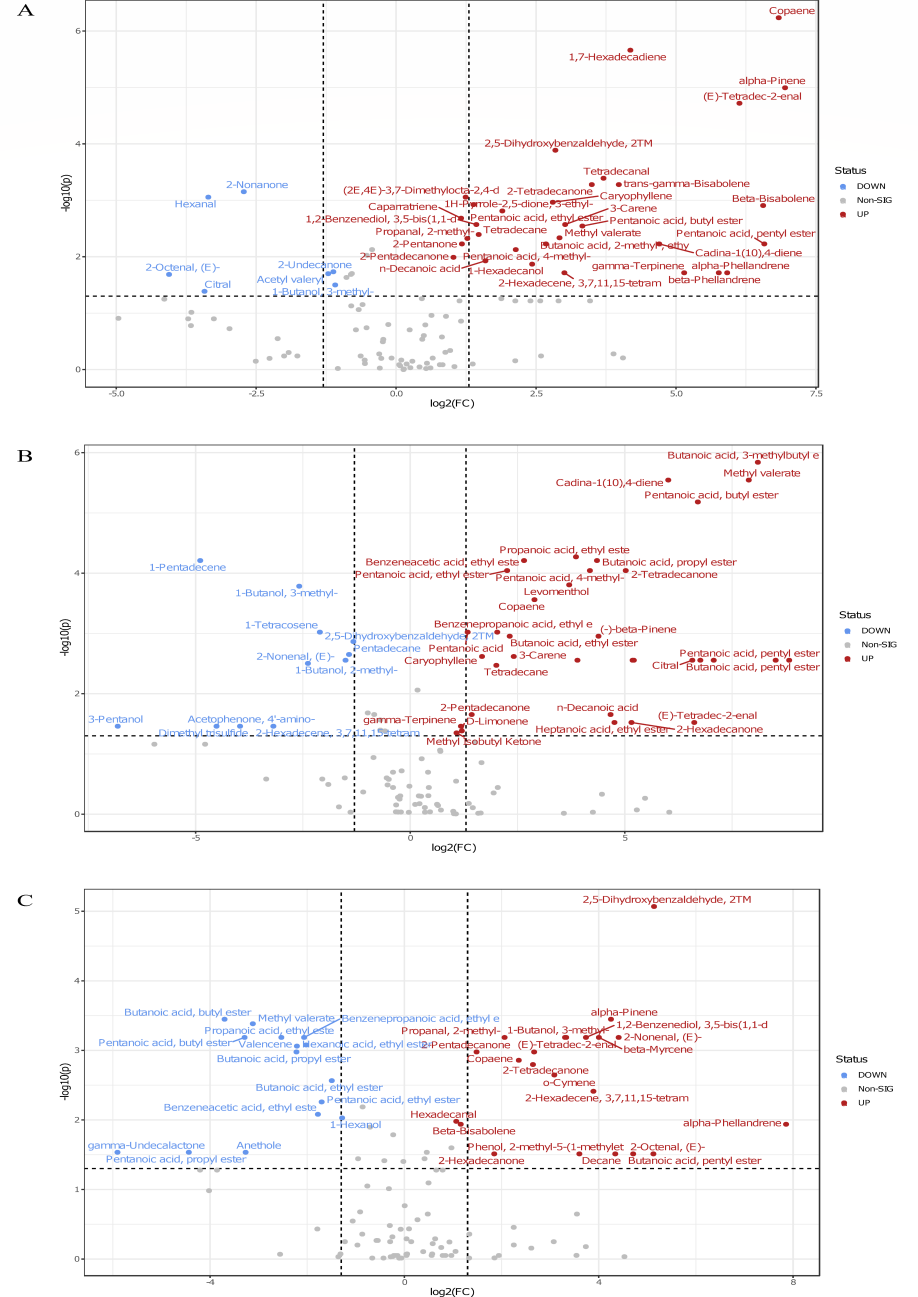
VOC volcano plot. Statistically significant VOC emerged from non-parametric Wilcoxon rank-sum test combined with FC analysis. Because of the chosen comparison direction of placebo/probiotic, increased and decreased metabolite concentrations in the placebo group were marked as down (blue) and up (red), respectively. The −log10 (*P*-values) is meaningful for the level of significance of each VOC and was plotted versus the log2 fold change. It represents the difference between the levels of expression for each VOC between the two groups at T1 (10 days of GFD) panel A, T5 panel B, and T6 panel C (20 days of 10 g/day gluten intake, of which the last 10 days were the wash-out). Pairwise comparison analysis based on the non-parametric Wilcoxon rank-sum test combined with FC was applied to evaluate differences between the levels of expression for each VOC between the two groups at T1, T5, and T6.

**Fig 8:**
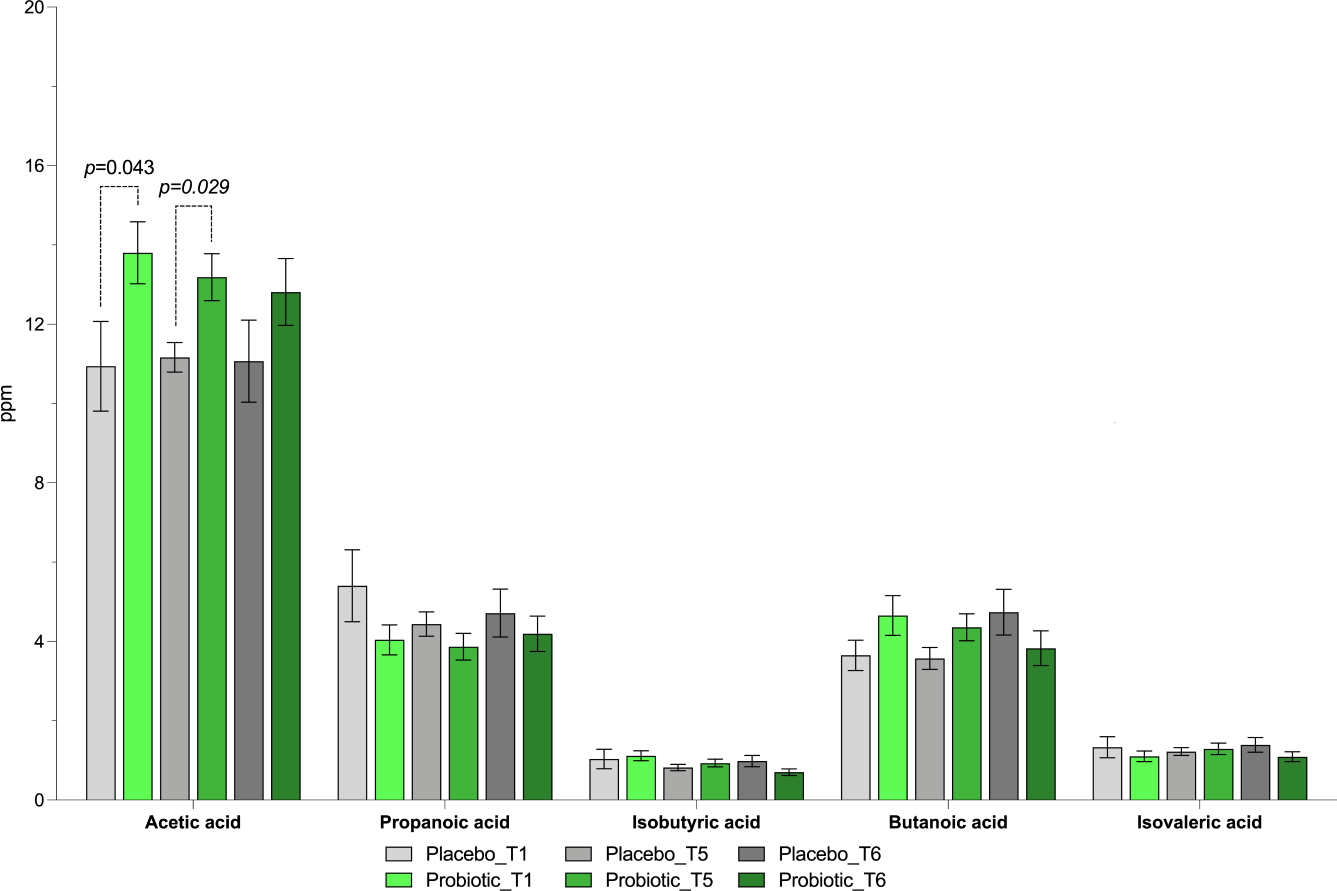
Fecal SCFAs and BCFAs. Concentration (ppm) of fecal SCFA and BCFA in the placebo and probiotic groups after 10 days of GFD (**T1**) and 20 days of 10 g/day gluten intake (**T5**), of which the last 10 days were the wash-out (**T6**). Adjusted *P*-values were obtained by the Kruskal–Wallis test corrected by Dunn’s multiple test.

Feces were analyzed for volatile compound (VOC) profile at T1, T5, and T6. Based on qualitative and quantitative differences, metabolomes were compared between the probiotic (*n* = 29) and placebo (*n* = 14) groups. One hundred and seventeen volatile metabolites were identified and classified as alcohols (*n* = 12), esters (*n* = 20), aldehydes (*n* = 11), phenols (*n* = 5), ketones (*n* = 13), organic acids (*n* = 13), terpenes (*n* = 18), hydrocarbons (*n* = 12), indoles (*n* = 2), lactones (*n* = 2), sulphur compounds (*n* = 2), and furans (*n* = 1). The supervised partial least squares-discriminant analysis (PLS-DA) was used for comparison (Fig. 6). The performance of PLS-DA models, including accuracy, goodness of fit (R2), and goodness of prediction (Q2), was assessed (Fig. 6A). For all comparisons, accuracy and R2 values were positive, and the higher value was recorded on the third component. A positive goodness of predictive ability (Q2) resulted in all models. Discriminant variables between groups were six compounds, with a variable importance in projection (VIP) score threshold of 1 (Fig. 6B). Skatole (3-methyl-indole) and hexanoic acid were higher in the probiotic group, while indole, *p*-cresol, caryophyllene, and pentanoic acid were relatively higher in the placebo group (Fig. 6C). We ran a pairwise non-parametric test to improve the inspection of those VOC, which allowed the two-group separation at each time point. More specifically, the volcano plot shown in Fig. 7 reports statistically significant VOCs, which emerged from a non-parametric Wilcoxon rank-sum test comparison vs a fold change (FC) analysis. Given our comparison direction of placebo versus probiotic, we designated increased and decreased metabolite concentrations in the placebo group as “up” (red) and “down” (blue), respectively (Fig. 7). At T1, seven compounds (2-nonanone, hexanal, 2-undecanone, acetyl valeryl, 2-octenal, (e)-, 1-butanol, 3-methyl-, and citral) were the most abundant in volunteers feed with probiotics. Compared to the probiotic group, 32 compounds were statistically significantly higher with placebo (Fig. 7A). At T5, the probiotic group was characterized by a higher concentration (*P* < 0.05, FC > 2) of 1-pentadecene, 3-methyl-1-butanol, 1-tetracosene, 2,5-dihydroxybenzaldehyde, pentadecane, 2-methyl-1-butanol, (e)-2-nonenal, 3-pentanol, dimethyl trisulfide, 4′-amino-acetophenone, and 3,7,11,15-tetramethyl-2-hexadecene (Fig. 7B). At T6, some other differences were raised from the pairwise comparison of the two groups (Fig. 7C). FC and *P*-values for all the statistically significant metabolites are reported in Table S2 to S4. We applied a Kruskal–Wallis (KW) test corrected by Dunn’s test to statistically assess the differences between the two groups at three sampling times. Several compounds differed (*P* < 0.05) along the probiotic treatment, including alcohols (e.g., phenylethyl alcohol), esters, medium- and long-chain fatty acids, and terpenes (Table S5). Four VOCs differed throughout the placebo treatment: ethyl esters of propanoic and pentanoic acids, propyl ester of butanoic acid, and beta-myrcene (Table S6). Targeted analyses of short-chain fatty acids (SCFAs) and branched-chain fatty acids (BCFAs) were carried out. Compared to the placebo group, the total concentration of SCFA (acetic, propionic, and butyric acids) was higher in the probiotic group at T1 (22.5 ± 1.66 vs 19.9 ± 1.4 ppm) and T5 (21.4 ± 1.27 vs 19.2 ± 0.91 ppm). Statistically significant differences based on pairwise comparison emerged. In particular, the highest concentration of acetic acid was detected in the probiotic group at T1 and T5 (Fig. 8). Butanoic and propanoic acids did not show statistically significant differences, similarly to isobutyric and isovaleric acids.

## DISCUSSION

Altogether gluten-related disorders show an increasing global trend, with a prevalence of approximately 5% of the worldwide population (6). Gluten is not fully digested in healthy individuals, but derived immunogenic epitopes promote disorders in predisposed people (13, 30). Compliance with GFD so far is the most efficient way to treat CD and is also the most recommended therapy for other gluten-related disorders. Nevertheless, the complete removal of gluten from the diet is arduous if not impossible (31, 32). Ingestion of gluten from cross-contaminated food even when following a GFD exceeds 50 mg per day. Even though chronic ingestion of minimal amounts of gluten (up to 50 mg/day) typically does not induce relapse of symptoms in CD patients, chronic ingestion of larger amounts (e.g., 100 mg/day) of gluten leads to a dose-dependent relapse of symptoms, which ranges from minimal morphometric changes of the jejunal histology to severe abnormalities of the mucosa. Although a clinical improvement under GFD is generally observed, mucosal abnormalities may persist but do not add up to functional malabsorption as clinical symptoms decline (10).

In theory, probiotics are a promising strategy for gluten detoxification, in the sense of a synergistic interaction between bacteria and gluten, as recently described (13). Three main mechanisms are conceivable: hydrolysis of gluten into small non-immunogenic polypeptides, limited access of immunogenic polypeptides to the lamina propria and reduced epithelial permeability, and maintenance of the gut microbiota homeostasis, with regulation of both internal and adaptive immune systems (11). To the best of our knowledge, our present study is the only randomized, placebo-controlled trial applying a synergistic consortium of probiotic bacteria for gluten degradation, with positive repercussions for not only gluten-related disorders but also enhancing the overall digestion of this unusual protein in healthy individuals. Similarly to our study, healthy volunteers are strongly recommended for phase one of clinical trials. This prevents risks of severe symptoms in CD patients and sets the baseline for subsequent studies (33). Despite the individual variability of the gluten fecal concentration at baseline, we showed that GFD compliance for 10 days was adequate to remove gluten residues from all feces of healthy volunteers. The intake of 50 mg of gluten per day for 4 consecutive days resulted in two and one positive volunteers for the probiotic (*n* = 50) and placebo (*n* = 20) groups. Previously (34), it was shown that micro-doses from 50 mg to 1 g of gluten/day made the gluten detectable in feces. Furthermore, the intake of 9 g gluten/day (4 days) up to 30 g gluten/day (other 4 days) positively correlated with the concentration of peptide derivatives in the feces. We observed the same trend, which was helpful in highlighting significant differences between the probiotic and placebo groups. At T4 (3 g gluten/day) and T5 (10 g gluten/day), the probiotic group showed much lower amounts of residual gluten in the feces. In another study without the administration of probiotics (35), healthy individuals were subjected to GFD and further administration of increasing amounts of gluten (50 mg and 2 g as single doses). A positive correlation between gluten intake and fecal content of immunogenic peptides was also confirmed. We daily administered gluten, and feces were collected at the end of each administration time, which lasted 4 days. Consequently, we postulated that false negatives were markedly limited, and the frequency of feces collection was adequate. After 10 days of wash-out, the gluten-degrading efficiency of the probiotic preparation seemed to decline, and no significant difference was observed between the intervention groups. This might be attributed to various complementary causes such as the reduced number of persisting probiotics, the loss of viability of some members from the probiotic preparation, the lack of protease and cytoplasmic extracts, and the consequent decrease of the synergistic effect (36).

Several reports already postulated the potential of probiotics to degrade gluten, but only in animal studies (37), using sourdough bread and not probiotics (38), or probiotic multi-species preparation for modulating the gut microbiota of celiac volunteers solely (39). Most of the *in vitro* findings referred to strains and species of lactobacilli, which were claimed as the main gluten-metabolizing bacteria in the gut (40, 41). Recently, the emerging application of probiotics also involves engineered synthetic probiotics (42). Nevertheless, the strength and novelty of our probiotic preparation lie in the complementarity of a multiple gluten-targeting action, which results from the complementary presence of selected lactic acid bacteria (*Lp. plantarum* DSM33363 and DSM33364*, Lc. paracasei* DSM33373*,* and *Ls. reuteri* DSM33374), spore-forming *Bacillus* (*B. megaterium* DSM33300 and *B. pumilus* DSM33297 and DSM33355), *Bacillus* protease, and lactic acid bacteria cytoplasmatic extracts. Previously, fungal proteases, in combination with lactic acid bacteria, contributed to the complete degradation of gluten during long-time sourdough fermentation (residual gluten less than 10 ppm) (43). *Bacillus* sp. with complementary and intense proteolytic activities and cytoplasmic extracts from lactic acid bacteria, to provide readily available peptidases, further enhanced the hydrolyzing power of our probiotic preparation (8). Overall, a multi-species probiotic mixture is more recommendable since the capability of probiotics to hydrolyze gluten epitopes is not found in single strains, species, or genera (44). The only previous report, which is in part comparable to our study, was the one providing the administration of an *Aspergillus niger*-derived protease to degrade gluten upon ingestion (45). As far as we know, this study did not proceed with a pharmaceutical application, and the stability of the enzyme during gastrointestinal digestion was questionable as well as its capability to consistently degrade gluten.

Numerous intervention studies (46) assessed probiotics or probiotic-added fermented foods for their capability to alter and colonize the gut microbiota of individuals, whether they were healthy or suffering from a disease. In our study, *Lachnospiraceae* and *Ruminococcaceae* were the most abundant families, which dominated the gut microbiota of volunteers belonging to both probiotic and placebo groups. Usually, the consistent presence of these families was related to a presumptive healthy microbiota (47). The third most abundant family was *Bifidobacteriaceae,* which was claimed for various potentialities, including the protection of the gut barrier functions (48). The gut microbiota of the two probiotic and placebo groups differed at the genus level. Volunteers fed with probiotics showed *Blautia*, *Faecalibacterium,* and *Bifidobacterium* as part of the topmost abundant taxa. In volunteers fed with placebo, *Agathobacter* replaced *Bifidobacterium*. Overall, *Blautia* and *Faecalibacterium* were associated with anti-inflammatory effects and with dietary habits rich in dietary fibers, vegetal proteins, and potassium and keto-analogs, which mimic the Mediterranean diet (49). The high adherence to the Mediterranean diet was the pre-requisite to recruiting volunteers for our study. The prevalence of *Bifidobacterium* might be somewhat related to the administration of probiotic lactic acid bacteria. Previously, the consumption of probiotic milk fermented with *Lactobacillus* was associated with higher levels of *Bifidobacterium* in the feces of healthy adults (50). Under gut homeostasis conditions, lactobacilli and *Bifidobacterium* were stable components of the intestinal microbiota, while mutually reduced abundances induced major depressive disorders (51). Other double-blind placebo-controlled studies with the administration of probiotic lactobacilli and/or bifidobacteria also showed significant differences in species counts and richness (alpha diversity) (52, 53). When we considered the whole intervention time and wash-out, we found the same. Similarly to another report (54), we did not find differences in alpha diversity when longitudinal sampling was compared for the same individual within each group (placebo or probiotic). Nevertheless, PERMANOVA showed that communities were slightly different between treatments (*P* = 0.001). Within each treatment (probiotic or placebo), there was also a significant difference (*P* = 0.011 and *P* = 0.043, respectively) for microbial communities between time points. LEfSe analysis demonstrated differentially abundant genera between placebo and probiotic interventions. Even though LEfSe analysis did not depict the genera included in the probiotic preparation as differentially abundant between interventions, other genera such as *Streptococcus* and *Lactococcus* were proacted by probiotic administration. Usually, it was speculated that these genera have positive effects on host health and high adaptability to gastrointestinal conditions (55, 56). Compared to healthy individuals, most of the duodenal biopsies from CD patients showed dysbiosis, with increased numbers of Gram-negative bacteria and decreased *Bifidobacterium*, *Streptococcus*, *Prevotella,* and *Lactobacillus* sp. (11). Our probiotics seemed to proact beneficial bacterial groups, which are essential to maintain gut homeostasis, and, in the case of *Streptococcus*, may show potential for synergistic degradation of gluten (16). The differentially abundant genera *Blautia*, *Oscillibacter,* and *Flavonifractor* found for the placebo group were reported as frequent inhabitants of the adult gut microbiota. Recent evidences placed *Blautia* as a new functional genus with potential probiotic properties (57), which was also one of the topmost abundant genera found for the probiotic group. Relative abundances of genera included in the probiotic preparation were all higher in the feces of volunteers fed with the preparation, with the most significant values for *Lactiplantibacillus* (*P* < 0.001) and *Limosilactobacillus* (*P* = 0.012). These results agreed with other studies that also observed a relative increase in the probiotic genera during the intervention (58, 59). Strict adherence to a GFD may result in a reduced intake of prebiotics, such as fructans and arabinoxylans, which impact the gut microbiota, revealing a detrimental effect on the microbial communities in both individuals with gluten-related disorders and healthy subjects (60). We sought to evaluate the effect of treatment on probiotic strains starting from a baseline condition in which there were no differences in terms of gluten intake that could reduce the density of health-promoting bacteria (61). Therefore, we quantitatively inspected the persistence of probiotic strains by RT-PCR at T1, T5, and T6. After wash-out, values of CN for *Ls. reuteri*, *Lc. paracasei*, and *B. magaterium* were significantly decreased compared to T5 but not with respect to T1, while CN for *Lp. plantarum* and *B. pumilus* maintained similar viabilities between T5 and T6, which might suggest an eventual intestinal colonization. Because of colonization resistance, most probiotics are excreted from the colon with feces after oral administration and soon after consumption ceases, making the probiotics undetectable. The mechanisms that cause colonization resistance can be divided into two broad categories: direct and indirect mechanisms (62). Direct colonization resistance is caused by the restriction of exogenous microbial colonization solely through factors associated with the gut microbiota, without any interaction with the host, and includes inhibition and resource competition (63). Indirect colonization resistance is dependent on host-derived factors such as antimicrobial peptide production, epithelial barrier maintenance, and bile acid concentration modulation via host interaction (64). Further, we should not overlook the fact that many of the probiotic benefits of probiotics might be gained from their dead cells as well (62).

With the aim of ascertaining the impact of probiotic treatment on human gut microbiota functionality, we investigated the fecal metabolome. The supervised PLS-DA revealed indoles (indole and 3-methyl-indole) and phenols (*p*-cresol) as the main contributing variables (VIP > 1). Usually, indoles and phenols are derived from the intestinal microbial activity toward aromatic amino acids (ArAAs) such as tryptophan (Trp), tyrosine, and phenylalanine (65). Intestinal bacteria convert Trp into tryptamine and indole-3-pyruvic acid and subsequently transform this latter into indole, indole-3-acetaldehyde, and indole lactic acid. Few species belonging to Firmicutes phyla (e.g., *Lactobacillus johnsonii, Ls. reuteri, Ligilactobacillus murinus,* and *Lactobacillus acidophilus*) have the capability to convert indole-3-acetaldehyde into indole-3-acetic acid and indirectly into 3-methyl-indole via decarboxylation. *Ls. reuteri* is one of the members of our probiotic preparation, and its abundance might explain the increased concentrations of 3-methyl-indole, which was found in the feces of volunteers fed with probiotics (66). Other Trp metabolites are generated by cooperating intestinal bacteria. For instance, the indole synthesis is catalyzed by tryptophanase activity from Firmicutes members such as *Enterobacter aerogenes* and *Clostridium* and some Bacteroidetes members, likely Fusobacteria and Proteobacteria (67). Microbial metabolites such as Trp were identified as emerging key players in host–gut microbiota cross-talk, acting as aryl hydrocarbon receptor (AhR) ligands and agonists (68, 69). AhR signaling initiates a cascade of immunomodulatory responses at the level of the gut mucosa barrier, including the modulation of intraepithelial lymphocytes, T cells, and group 3 innate lymphoid cells, which synthesize interleukin-17 (IL-17) and IL-22. All these responses are beneficial to gluten-related disorders (70). Aryl hydrocarbon receptor expression is down-regulated in inflamed mucosa of CD patients (71). It was proposed that the activation of AhR by commensal bacteria such as *Lactobacillus* and *Bifidobacterium* might be a new therapeutic target in the modulation of human intestinal inflammation (72). Although indole might act as an AhR ligand, it is also a precursor of uremic toxins along with *p*-cresol (73). *P*-cresol is another product from ArAA metabolism, which *in vitro* decreased the intestinal epithelial barrier function and acted as a presumptive carcinogenic compound (74). The lowest concentration of *p*-cresol in the feces from volunteers fed with probiotics might be somewhat related to the abundance of lactic acid bacteria that tolerated this compound (75). SCFAs are end-products of intestinal fermentation that are used both as energy sources for colon cells or biogenic compounds for immune regulation and intestinal barrier function (58). The statistically significant increase of acetic acid in the feces of volunteers fed with probiotics at T1 and T5 would suggest a contribution of the treatment in immune cell responses. Very little information is available in the literature concerning microbial pathways responsible for the synthesis of volatile esters at the level of the human gut. At T1 and T5, we showed decreased concentrations of some carboxylic acid esters for probiotic with respect to the placebo group. Bacterial esterases catalyzed esterification reactions of such organic acids and alcohols (76), which might act as sources of esters. Besides, esters are synthesized by non-enzymatic esterification reactions that involve gut luminal or bacterial substrates.

### Conclusions

In conclusion, our clinical trial provides novel insights into the gluten-hydrolyzing capability of our probiotic preparation (In Vivo Biotics gluten tolerance). While the findings are promising, a cautious interpretation is warranted, considering our study is in the early phase (phase 0), and further research with larger sample sizes is required. The observed positive impact on gut microbiota richness and potential immunomodulatory effects hint at additional potential benefits for gluten-related disorders. However, validation is required in follow- up studies also involving participants with CD.

## MATERIALS AND METHODS

### Volunteers’ recruitment criteria

Recruitment concerned only healthy individuals. Women and men between 18 and 65 years old with a normal body mass index (BMI: 18.5–25 kg m^−2^) were recruited. The exclusion criteria were as follows: known medical disease, digestive disease symptoms and family history of CD, wheat allergy, and use of prescription medications (including antibiotics or probiotics in the previous 2 months). Dieticians and physicians cooperated with the Free University of Bolzano for individual anamneses, diet guideline, and recruitment. During recruitment, eligible individuals (satisfactory anamnesis) filled out a questionnaire dealing with their recurrent dietary habits. Based on the elaboration of these questionnaires, individuals were recruited only if adherent to the Mediterranean diet (Table S7). This selection eliminated confounding factors related to dietary habits. Volunteers meeting the eligibility criteria received comprehensive lists of gluten-free foods and those requiring careful examination before consumption, to adhere to GFD. The compilation of these lists and the development of suggested meals were meticulously crafted in alignment with the guidelines provided by the Associazione Italiana Celiachia (Supplementary Guidelines diet S1) (77). The potential risk of gluten cross-contamination from gluten-free products was evaluated during the first 10 days of the challenge in which participants followed strictly the GFD, and gluten residues were absent from their feces.

### Probiotic preparation

The probiotic composition (In Vivo Biotics gluten tolerance) comprised seven probiotic strains deposited in the Deutsche Sammlung von Mikroorganismen und Zellkulturen GmbH collection: *Lactiplantibacillus plantarum* DSM33363 (B4U33, Evonik, Darmstadt), *Lactiplantibacillus plantarum* DSM33364 (B4U64, Evonik, Darmstadt),

*Lacticaseibacillus paracasei* DSM33373 (B4U73. Evonik, Darmstadt), *Limosilactobacillus reuteri* DSM33374 (B4U74, Evonik, Darmstadt), *Bacillus megaterium* DSM33300 (B4U07, Evonik, Darmstadt), *Bacillus pumilus* DSM33297 (B4U97, Evonik, Darmstadt), and *Bacillus pumilus* DSM33355 (B4U55, Evonik, Darmstadt). Strains were meticulously chosen through an extensive screening process involving 504 strains, encompassing both lactic acid bacteria and *Bacillus* spp. Further insights into the selection criteria and evaluation can be found in our preceding study (8), providing detailed information on the process. All strains were evaluated for safety according to the Qualified Presumption of Safety (QPS) assessment (doi: 10.2903 /j.efsa.2017.4663) and complied with the QPS safety requirements. The formula also included freeze-dried cytoplasmic extracts (corresponding to cell biomass derived from ≥3 × 10^9^ CFU per capsule) from the above-mentioned probiotic strains (8) and 10 mg of a food-grade *Bacillus* protease preparation (Promod D24MDP, Biocatalysts). The probiotic preparation was provided by Evonik Operations GmbH (Germany) in the form of capsules, containing ≥3 × 10^9^ CFU of viable bacteria. The probiotic preparation was stored for up to 6 months at room temperature, and viable cell numbers, as determined by cultural enumeration, were shown to be stable throughout the entire study period (data not shown). The capsule material was hydroxypropyl methylcellulose.

### *In vivo* challenge and sampling

This study was a randomized, double-blinded, placebo-controlled *in vivo* challenge (Fig. 1). Volunteers were divided into two groups: 50 allocated to probiotic group administration and 20 to placebo. Most of the volunteers were male (*n* = 38) with an average age of 36 ± 12.28 years and an average BMI of 22.00 ± 1.68 kg m^−2^, while the female volunteers (*n* = 32) had an average age of 38 ± 12.00 years and an average BMI of 21.00 ± 1.50 kg m^−2^. Capsules (one probiotic or one placebo per day) were administered before the main meal (lunch) for a total of 32 days. Volunteers were provided with enough capsules for the entire length of the treatment period and were instructed to daily consume one capsule. To eliminate residual traces of gluten and similar proteins from the feces, both groups underwent a GFD from day 1 to day 10. After 10 days, still under GFD, gluten administration started. Gluten capsules were produced by Evonik Operations GmbH explicitly for the study. Given that cross-contamination of gluten on a GFD may vary from 5 to 50 mg per day, we opted to commence the challenge with the highest amount referenced in the literature. Chronic ingestion of larger amounts (e.g., 100 mg/day) of gluten leads to a dose-dependent relapse of CD symptoms, while 50 mg per day may not (10). Based on the above, the increasing administration plan was as follows: 50 mg/day for 4 days, 1 g/day for the subsequent 4 days, 3 g/day for the subsequent 4 days, and 10 g/day (in this case, reintroducing an equivalent amount of wheat-based bread—four slices) for the subsequent 20 days. At this stage (10 + 4 + 4 + 4 + 10 days = total of 32 days), the administration of the probiotic or placebo preparation was interrupted, with a period of 10 days of wash-out. The collection of feces was at baseline (T0), 10 days of GFD (T1), 4 days of 50 mg/day gluten intake (T2), 4 days of 1 g/day gluten intake (T3), 4 days of 3 g/day gluten intake (T4), and 20 days of 10 g/day gluten intake (T5), of which the last 10 days was the wash-out (T6).

Volunteers were instructed to collect feces in a sealed sterilized container, which was dropped off at the laboratory within 6 h of collection. To avoid freeze-thawing, each sample was separated into several sterile containers and stored at −80°C until further processing.

### Competitive ELISA

The ELISA Ridascreen Gliadin competitive kit (R7021, R-Biopharm, Italy) was used to detect residual gluten in feces. The analysis was according to the manufacturer’s instructions, with some modifications. After defrosting the feces, 1 g was dissolved in 10.0 mL of 60% (vol/vol) ethanol. The mixture was homogenized and incubated at 50°C for 1 h in a shaking incubator (150 rpm). Then, the sample was centrifuged (2,500 rpm) at room temperature for 10 min. The supernatant was filtered (0.22 µm membrane) and collected for further analysis. Samples were diluted 1:50, 1:100, 1:200, and 1:500 with dilution buffer according to the administered gluten concentrations. The analyses were in duplicates. The diluted sample (50 µL) and standards were added to the wells. Then, diluted conjugate (50 µL) was added to each well, after gently shaking the micro-titer plate. An incubation of 30 min at room temperature followed. Wells were washed four times with 250 µL of washing buffer. Subsequently, 100 µL of chromogen was added to wells, and the plate was incubated for 10 min at room temperature in the dark. In the end, 100 µL of stop solution was added, and after gently shaking, the absorbance was measured at 450 nm using an Infinite M Nano+ Spectrophotometer (Tecan, Austria). The Ridasoft Win.NET Food & Feed (version 1.2.1. beta) software was used for the result evaluation. The gliadin competitive kit used targets the R5 monoclonal antibody that recognizes potentially toxic peptide sequences of gliadins from wheat and related prolamins from rye and barley, which also can be found in gluten peptides resistant to gastrointestinal digestion. The hydrolyzed gluten was expressed in gliadin concentration (ng/mL) (ppb) from the standard curve and was further multiplied by the corresponding dilution factor. Then, the residual gluten content was determined by multiplying the gliadin fraction by a factor of 2 as recommended by the manufacturer. The limit of detection for gluten was 4.6 ppm, and the limit of quantification was 10 ppm. The kit’s validation involved using fecal samples with different concentrations of spiked gluten (ranging from 0 to 270 ng/mL) following the extraction procedure described earlier. The standard curve was constructed using cubic spline interpolation. In cases where data points exceeded the last point of the calibration curve, samples were appropriately diluted until they aligned with the calibration data. Data below the detection limit were replaced with zeros and were accounted for in the average determination and statistics.

### DNA metabarcoding sequencing and analyses

A longitudinal 16S rRNA metagenomic analysis was carried out on feces from a subset of volunteers (*n* = 29 and *n* = 14 for probiotic and placebo groups, respectively), who were randomly selected upon statistical evaluation. Feces were collected prior to probiotic consumption (T0; baseline) and at T1, T2, T3, T4, T5, and T6. Microbiome DNA was extracted from feces by the Spin Kit for Soil (MP Biomedicals, Italy). Primers targeting the 16S rRNA variable region V3-V4 (*Escherichia coli* position 341–805, forward 341F: CCTACGGGNGGCWGCAG and reverse 806R: GACTACNVGGGTWTCTAATCC) were used for library preparation. In the first amplification step, PCRs were carried out in a final volume of 12.5 µL, containing 1.25 µL of template DNA, 0.5 µM of the primers, 3.13 µL of Supreme NZYTaq 2× Green Master Mix (NZYTech), and ultrapure water up to 12.5 µL. The reaction mixture was incubated as follows: an initial denaturation step at 95°C for 5 min, followed by 25 cycles of 95°C for 30 s, 45°C for 45 s, 72°C for 45 s, and a final extension step at 72°C for 7 min. The oligonucleotide indices that were required for multiplexing different libraries in the same sequencing pool were attached in a second amplification step with identical conditions but only five cycles and 60°C as the annealing temperature. The library size was verified by running the libraries on 2% agarose gels stained with GreenSafe (NZYTech) and imaging them under UV light. Then, the libraries were purified using the Mag-Bind RxnPure Plus magnetic beads (Omega Bio-tek), following the instructions provided by the manufacturer. Finished libraries were pooled in equimolar amounts according to the results of a Qubit dsDNA HS Assay (Thermo Fisher Scientific) quantification. The pool was sequenced in a fraction of a MiSeq PE300 flow cell (Illumina) aiming for a total output of 0.5 gigabases per sample. Library preparation and pair-end sequencing were carried out at the Biomes.World (Berlin, Germany) using the Illumina MiSeq system (Illumina, USA). The quality of the FASTQ files was assessed with the software FastQC (78), and the output was summarized using MultiQC (79). The obtained amplicon reads were processed using QIIME 2 (release 2022.2) (80). Specifically, the tool DADA2 (81) (implemented in QIIME 2) was used to remove the PCR primers, filter the reads according to their quality, denoise, merge the forward and reverse reads, remove the chimeric reads, and cluster the resulting sequences into OTUs. To confirm the presence of the probiotic species in the fecal samples, we used BLAST on OTUs assigned to the *Lactobacillaceae* and *Bacillaceae* families. Confirmed genera were then aligned with BLAST against the reference genomes of the probiotic strains (>99% sequence similarity).

### Quantitative real-time PCR

qPCR was carried out to quantitatively assay probiotic strains from the same volunteers selected for DNA metabarcoding analysis (*n* = 29 and *n* = 14 for the probiotic and placebo groups, respectively). Sampling was at T1, T5, and T6. Total RNA was extracted from an aliquot of ca. 200 mg of feces using the RNeasy microbiome kit, according to the manufacturer’s instructions (Qiagen, Milano, Italy). DNA was extracted from 500 mg of feces. Each aliquot was twice washed with 1 mL PBS-EDTA (phosphate buffer 0.01 M, pH 7.2, 0.01 M EDTA) and centrifuged (14,000 × *g* at 4°C for 5 min) (82). The residual pellet was resuspended in 500 µL of PBS-EDTA, and the FastDNA Pro Soil-Direct Kit (MP Biomedicals, California, USA) was used for total DNA extraction. DNA extraction yield (ng/µL) and quality check (260/280 nm and 260/230 nm ratio) were measured spectrophotometrically with a NanoDrop 2000c Spectrophotometer (Thermo Fisher Scientific Inc., MI, Italy). RNA extraction yield and quality check were assessed under the above conditions. The cDNA was synthesized using 14.0 µL of the extracted RNA through the iScript gDNA Clear cDNA Synthesis Kit (# 172-5035, Bio-Rad). Specifically, *Lp. plantarum* DSM33363 and DSM33364, *Ls. reuteri* DSM33373, *Lc. paracasei* DSM33374, *B. megaterium* DSM33300, and *B. pumilus* DSM33355 and DSM33297 were investigated as both DNA and cDNA using different primers (27–29) (Table 1). The qPCR reactions were carried out with the Applied Biosystems 7300 Real-Time PCR System (Thermo Fisher Scientific Inc., MI, Italy). The total reaction mix (25 µL) contained 12.5 µL SYBR Green Mix (# 1725271, Bio-Rad Laboratories S.r.l., Milano, Italy), 0.1 µL primer (0.2 µM), 11.4 µL DNase- and RNase-free water, and 1.0 µL of template corresponding to 40 ng of DNA or cDNA. Each reaction was performed in triplicate. The amplification steps consisted of one cycle at 95°C for 3 min, 40 cycles at 95°C for 5 s, appropriate annealing temperatures (Table 1) for 40 s, and the final step at 72°C for 1 min. The PCR amplicon melting curve analysis started at a temperature of 60°C with increases of 1°C/5 s until the final temperature of 95°C. For each primer, the qPCR results (cycle threshold, C_T_) were converted in CN based on standard curves previously constructed by using serial dilutions of DNA extracted from pure cultures. The CN and CN(log) were calculated based on DNA concentration and amplicon length. The standard curves were obtained by C_T_ and CN(log) interpolation.

### Gas-chromatography mass spectrometry-solid-phase microextraction analysis

One gram of feces (*n* = 29 and *n* = 14 for the probiotic and placebo groups, respectively) was placed into 10-mL glass vials. Ten microliters of 4-methyl-2-pentanol (final concentration of 1 mg L^−1^) was added as an internal standard. Samples were equilibrated for 10 min at 60°C. Solid-phase microextraction fiber (divinylbenzene/Carboxen/polydimethylsiloxane) was exposed to each sample for 40 min. The VOCs were thermally desorbed by immediately transferring the fiber into the heated injection port (220°C) of Clarus 680 (Perkin Elmer, Beaconsfield, UK) gas chromatography equipped with an Rtx-WAX column (30 m × 0.25 mm i.d., 0.25 µm film thickness) (Restek) and coupled to a Clarus SQ8MS (Perkin Elmer) (83). The column temperature was set initially at 35°C for 8 min, then increased to 60°C at 4°C min^−1^, to 160°C at 6°C min^−1^, and finally to 200°C at 20°C min^−1^ and held for 15 min. Spitless injection was used for sample introduction into the capillary column. Helium was used as carrier gas with a flow rate of 1 mL min^−1^. The source and transfer line temperatures were maintained at 250°C and 230°C, respectively. Electron ionization masses were recorded at 70 eV in the mass-to-charge ratio interval, which was from 34 to 350 m/z. The gas chromatography–mass spectrometry generated a chromatogram with peaks representing individual compounds. Each chromatogram was analyzed for peak identification using the National Institute of Standard and Technology 2008 library. A peak area threshold of >1,000,000 and 85% or greater probability of match was used for VOC identification, followed by manual visual inspection of the fragment patterns when required. 4-Methyl-2-pentanol (final concentration 1 mg L^−1^) was used as an internal standard in all analyses, to quantify the identified compounds by interpolation of the relative areas versus the internal standard area.

### Short-chain fatty acid quantification

A stock solution containing the mixture of SCFA standards (acetic acid, butyric acid, propionic acid, isobutyric acid, and valeric acid) dissolved in ultrapure water was diluted to obtain a calibration curve ranging from 1 to 250 μg mL^−1^. Ten microliters of 4-methyl-2-pentanol (final concentration of 1 mg L^−1^) was added as an internal standard in each dilution, before the analysis. We used the total ion current mode to obtain typical ions with a special mass-to-charge ratio of every SCFA and then used a selective ion monitoring (SIM) mode to collect information on typical ions (84). A standard curve was made for each SCFA based on the data obtained by the SIM mode. The calibration curve was constructed by plotting the normalized peak area versus the concentration of individual SCFA. The relative peak of SCFA in feces was integrated, and the concentration of SCFA was calculated by the calibration curve equation.

### Basic statistical analyses and data integration

Statistical elaboration of residual gluten results was done with XLSTAT (v. 4.1). Statistical significance testing between the probiotic and placebo groups per time point was done by the Mann–Whitney test (exact *P* < 0.0001). For the microbiome (DNA metabarcoding), statistical analyses and plotting were done within the R environment (v. 4.2.2) (R core team, 2022) and the packages phyloseq (v. 1.40.0) (85), vegan (v.2.6.2) (86), rstatix (v. 0.7.0) (86), and ggplot2 (v. 3.3.6) (87). Alpha diversity measures were calculated on raw counts to capture the full diversity of the samples. The significance of richness between probiotic and placebo samples was calculated by the pairwise Wilcoxon rank-sum test. Beta diversity was calculated on taxonomic relative abundance, where OTUs were agglomerated on the genus level and genera with abundances <0.02% were removed. Differences in communities between treatment and time points were explored by a PCoA with Bray–Curtis dissimilarities. PERMANOVA on the distance matrix with the adonis function in vegan was performed to test if there were differences between the groups (treatments and time points). We used LEfSe (88) to determine taxa that were differently abundant between treatments and time points. Differences in abundances of the probiotic taxa (*Lp. plantarum* DSM33363 and DSM33364, *Lc. paracasei* DSM33373, *Ls. reuteri* DSM33374, *B. megaterium* DSM33300, and *B. pumilus* DSM33335 and DSM33297) during and after treatment (T1–T6) were tested between the probiotic and placebo samples (Wilcoxon rank-sum test). The dynamics of each taxa were calculated by conducting Kruskal–Wallis tests and pairwise Wilcoxon rank-sum test between T0 and each subsequent time point (T1–T6). Multiple testing was adjusted with the Benjamini–Hochberg method. Bar plots (mean ± standard deviation) were used to elaborate and compare the qPCR results of specific time points (T1, T5, and T6) of both probiotic and placebo groups. Two-tailed Student’s *t*-test was carried out to compare mean values, and significance was at *P*-values <0.05. The supervised PLS-DA of VOC profiles was used to compare intervention groups, while statistically significant differences within each intervention group for VOC, targeted SCFA, and BCFA at three sampling times (T1, T5, and T6) were estimated using the KW test corrected by Dunn’s test. A pairwise comparison analysis based on the non-parametric Wilcoxon rank-sum test combined with FC was applied to evaluate differences between the levels of expression for each VOC between the two groups at T1, T5, and T6.

## Data Availability

16S rRNA gene amplicon sequencing data were deposited under BioProject accession number PRJNA941234.

## SUPPLEMENTAL MATERIAL

Supplemental figures spectrum.03524-23-s0001.pdf Fig. S1-S3.

Supplemental guidelines diet spectrum.03524-23-s0002.pdf Guidelines for a correct gluten free diet.

Table S1 spectrum.03524-23-s0003.docx Differentially abundant taxa.

Table S2 spectrum.03524-23-s0004.docx Volcano plot results T1.

Table S3 spectrum.03524-23-s0005.docx Volcano plot results T5.

Table S4 spectrum.03524-23-s0006.docx Volcano plot results T6.

Table S5 spectrum.03524-23-s0007.docx Fecal VOC probiotic group.

Table S6 spectrum.03524-23-s0008.docx Concentration (ppm) of statistically significant fecal VOC.

Table S7 spectrum.03524-23-s0009.docx Mediterranean Diet Serving Score.

ASM does not own the copyrights to Supplemental Material that may be linked to, or accessed through, an article. The authors have granted ASM a non-exclusive, world-wide license to publish the Supplemental Material files. Please contact the corresponding author directly for reuse.

## References

[1] Wei G, Helmerhorst EJ, Darwish G, Blumenkranz G, Schuppan D. 2020. Gluten degrading enzymes for treatment of celiac diseas. Nutrients 12:2095. doi:10.3390/nu12072095 32679754 PMC7400306

[2] Wieser H. 2007. Chemistry of gluten proteins. Food Microbiol 24:115–119. doi:10.1016/j.fm.2006.07.004 17008153

[3] Biesiekierski JR. 2017. What is gluten? J Gastroenterol Hepatol 32 Suppl 1:78–81. doi:10.1111/jgh.13703 28244676

[4] Algera JP, Störsrud S, Lindström A, Simrén M, Törnblom H. 2021. Gluten and fructan intake and their associations with gastrointestinal symptoms in irritable bowel syndrome: a food diary study. Clin Nutr 40:5365–5372. doi:10.1016/j.clnu.2021.09.002 34560607

[5] Caio G, Volta U, Sapone A, Leffler DA, De Giorgio R, Catassi C, Fasano A. 2019. Celiac disease: a comprehensive current review. BMC Med 17:142. doi:10.1186/s12916-019-1380-z 31331324 PMC6647104

[6] Taraghikhah N, Ashtari S, Asri N, Shahbazkhani B, Al-Dulaimi D, Rostami-Nejad M, Rezaei-Tavirani M, Razzaghi MR, Zali MR. 2020. An updated overview of spectrum of gluten-related disorders: clinical and diagnostic aspects. BMC Gastroenterol 20:258. doi:10.1186/s12876-020-01390-0 32762724 PMC7409416

[7] Cristofori F, Indrio F, Miniello VL, De Angelis M, Francavilla R. 2018. Probiotics in celiac disease. Nutrients 10:1824. doi:10.3390/nu10121824 30477107 PMC6316269

[8] De Angelis M, Siragusa S, Vacca M, Di Cagno R, Cristofori F, Schwarm M, Pelzer S, Flügel M, Speckmann B, Francavilla R, Gobbetti M. 2021. Selection of gut-resistant bacteria and construction of microbial consortia for improving gluten digestion under simulated gastrointestinal conditions. Nutrients 13:992. doi:10.3390/nu13030992 33808622 PMC8003469

[9] Al-Sunaid FF, Al-Homidi MM, Al-Qahtani RM, Al-Ashwal RA, Mudhish GA, Hanbazaza MA, Al-Zaben AS. 2021. The influence of a gluten-free diet on health-related quality of life in individuals with celiac disease. BMC Gastroenterol 21:330. doi:10.1186/s12876-021-01908-0 34433427 PMC8390240

[10] Ullrich R. 2019. Gluten-free diet in celiac disease—forever and for all? Nutrients. doi:10.3390/nu10111796 PMC6267495 30453686

[11] Chibbar R, Dieleman LA. 2019. The gut microbiota in celiac disease and probiotics. Nutrients 11:2375. doi:10.3390/nu11102375 31590358 PMC6836185

[12] Kõiv V, Tenson T. 2021. Gluten-degrading bacteria: availability and applications. Appl Microbiol Biotechnol 105:3045–3059. doi:10.1007/s00253-021-11263-5 33837830 PMC8053163

[13] Speckmann B, Ehring E, Hu J, Rodriguez Mateos A. 2024. Exploring substrate–microbe interactions: a metabiotic approach toward developing targeted synbiotic compositions. Gut Microbes 16:2305716. doi:10.1080/19490976.2024.2305716 38300741 PMC10841028

[14] Moreno MD, Rodríguez-Herrera A, Sousa C, Comino I. 2017. Biomarkers to monitor gluten-free diet compliance in celiac patients. Nutrients 9:46. doi:10.3390/nu9010046 28067823 PMC5295090

[15] Pecora F, Persico F, Gismondi P, Fornaroli F, Iuliano S, de’Angelis GL, Esposito S. 2020. Gut microbiota in celiac disease: is there any role for probiotics? Front Immunol 11:957. doi:10.3389/fimmu.2020.00957 32499787 PMC7243837

[16] Herrán AR, Pérez-Andrés J, Caminero A, Nistal E, Vivas S, Ruiz de Morales JM, Casqueiro J. 2017. Gluten-degrading bacteria are present in the human small intestine of healthy volunteers and celiac patients. Res Microbiol 168:673–684. doi:10.1016/j.resmic.2017.04.008 28526528

[17] Caminero A, Galipeau HJ, McCarville JL, Johnston CW, Bernier SP, Russell AK, Jury J, Herran AR, Casqueiro J, Tye-Din JA, Surette MG, Magarvey NA, Schuppan D, Verdu EF. 2016. Duodenal bacteria from patients with celiac disease and healthy subjects distinctly affect gluten breakdown and immunogenicity. Gastroenterology 151:670–683. doi:10.1053/j.gastro.2016.06.041 27373514

[18] De Angelis M, Cassone A, Rizzello CG, Gagliardi F, Minervini F, Calasso M, Francavilla R, Gobbetti M. 2010. Mechanism of degradation of immunogenic gluten epitopes from Triticum turgidum L. var. durum by sourdough lactobacilli and fungal proteases. Appl Environ Microbiol 76:508–518. doi:10.1128/AEM.01630-09 19948868 PMC2805216

[19] Marasco G, Cirota GG, Rossini B, Lungaro L, Di Biase AR, Colecchia A, Volta U, De Giorgio R, Festi D, Caio G. 2020. Probiotics, prebiotics and other dietary supplements for gut microbiota modulation in celiac disease patients. Nutrients 12:2674. doi:10.3390/nu12092674 32887325 PMC7551848

[20] Francavilla R, De Angelis M, Rizzello CG, Cavallo N, Dal Bello F, Gobbetti M. 2017. Selected probiotic lactobacilli have the capacity to hydrolyze gluten peptides during simulated gastrointestinal digestion. Appl Environ Microbiol 83:e00376–17. doi:10.1128/AEM.00376-17 28500039 PMC5494637

[21] Greco L, Gobbetti M, Auricchio R, Di Mase R, Landolfo F, Paparo F, Di Cagno R, De Angelis M, Rizzello CG, Cassone A, Terrone G, Timpone L, D’Aniello M, Maglio M, Troncone R, Auricchio S. 2011. Safety for patients with celiac disease of baked goods made of wheat flour hydrolyzed during food processing. Clin Gastroenterol Hepatol 9:24–29. doi:10.1016/j.cgh.2010.09.025 20951830

[22] Dumville JC, Hahn S, Miles JNV, Torgerson DJ. 2006. The use of unequal randomisation ratios in clinical trials: a review. Contemp Clin Trials 27:1–12. doi:10.1016/j.cct.2005.08.003 16236557

[23] Tremblay A, Fatani A, Ford AL, Piano A, Nagulesapillai V, Auger J, MacPherson CW, Christman MC, Tompkins TA, Dahl WJ. 2021. Safety and effect of a low- and high-dose multi-strain probiotic supplement on microbiota in a general adult population: a randomized, double-blind, placebo-controlled study. J Diet Suppl 18:227–247. doi:10.1080/19390211.2020.1749751 32306803

[24] Botelho PB, Ferreira MVR, Araújo A de M, Mendes MM, Nakano EY. 2020. Effect of multispecies probiotic on gut microbiota composition in individuals with intestinal constipation: a double-blind, placebo-controlled randomized trial. Nutrition 78:110890. doi:10.1016/j.nut.2020.110890 32738573

[25] Monteagudo C, Mariscal-Arcas M, Rivas A, Lorenzo-Tovar ML, Tur JA, Olea-Serrano F. 2015. Proposal of a mediterranean diet serving score. PLoS One 10:e0128594. doi:10.1371/journal.pone.0128594 26035442 PMC4452755

[26] Bach-Faig A, Berry E, Lairon D, Reguant J, Trichopoulou A, Dernini S, Medina FX, Battino M, Belahsen R, Miranda G, Serra-Majem L. 2011. Mediterranean diet foundation expert group, mediterranean diet pyramid today. Science and cultural updates. Public Health Nutr 14:2274–2284. 22166184 10.1017/S1368980011002515

[27] Jomehzadeh N, Javaherizadeh H, Amin M, Rashno M, Teimoori A. 2020. Quantification of intestinal Lactobacillus species in children with functional constipation by quantitative real-time PCR. Clin Exp Gastroenterol 13:141–150. doi:10.2147/CEG.S250755 32440191 PMC7211309

[28] Nayak PK, Mohanty AK, Gaonkar T, Kumar A, Bhosle SN, Garg S. 2013. Rapid identification of polyhydroxyalkanoate accumulating members of bacillales using internal primers for phaC gene of Bacillus megaterium. ISRN Bacteriol 2013:1–12. doi:10.1155/2013/562014

[29] Khowal S, Siddiqui MZ, Ali S, Khan MT, Khan MA, Naqvi SH, Wajid S. 2017. A report on extensive lateral genetic reciprocation between arsenic resistant Bacillus subtilis and Bacillus pumilus strains analyzed using RAPD-PCR. Mol Phylogenet Evol 107:443–454. doi:10.1016/j.ympev.2016.12.010 27956257

[30] Shan L, Molberg Ø, Parrot I, Hausch F, Filiz F, Gray GM, Sollid LM, Khosla C. 2002. Structural basis for gluten intolerance in celiac sprue. Science 297:2275–2279. doi:10.1126/science.1074129 12351792

[31] Leonard MM, Sapone A, Catassi C, Fasano A. 2017. Celiac disease and nonceliac gluten sensitivity: a review. JAMA 318:647–656. doi:10.1001/jama.2017.9730 28810029

[32] Heredia-Sandoval NG, Valencia-Tapia MY, Calderón de la Barca AM, Islas-Rubio AR. 2016. Microbial proteases in baked goods: modification of gluten and effects on immunogenicity and product quality. Foods 5:59. doi:10.3390/foods5030059 28231153 PMC5302405

[33] FDA. 2018. Step 3: clinical trials. Available from: https://www.fda.gov/patients/drug-development-process/step-3-clinical-research

[34] Comino I, Real A, Vivas S, Síglez MÁ, Caminero A, Nistal E, Casqueiro J, Rodríguez-Herrera A, Cebolla A, Sousa C. 2012. Monitoring of gluten-free diet compliance in celiac patients by assessment of gliadin 33-mer equivalent epitopes in feces. Am J Clin Nutr 95:670–677. doi:10.3945/ajcn.111.026708 22258271 PMC3278243

[35] Coto L, Sousa C, Cebolla A. 2022. Individual variability in patterns and dynamics of fecal gluten immunogenic peptides excretion after low gluten intake. Eur J Nutr 61:2033–2049. doi:10.1007/s00394-021-02765-z 34993643 PMC8739026

[36] Toscano M, De Grandi R, Pastorelli L, Vecchi M, Drago L. 2017. A consumer’s guide for probiotics: 10 golden rules for a correct use. Dig Liver Dis 49:1177–1184. doi:10.1016/j.dld.2017.07.011 28830747

[37] D’Arienzo R, Maurano F, Lavermicocca P, Ricca E, Rossi M. 2009. Modulation of the immune response by probiotic strains in a mouse model of gluten sensitivity. Cytokine 48:254–259. doi:10.1016/j.cyto.2009.08.003 19736022

[38] Di Cagno R, De Angelis M, Auricchio S, Greco L, Clarke C, De Vincenzi M, Giovannini C, D’Archivio M, Landolfo F, Parrilli G, Minervini F, Arendt E, Gobbetti M. 2004. Sourdough bread made from wheat and nontoxic flours and started with selected lactobacilli is tolerated in celiac sprue patients. Appl Environ Microbiol 70:1088–1096. doi:10.1128/AEM.70.2.1088-1096.2004 14766592 PMC348803

[39] Francavilla R, Piccolo M, Francavilla A, Polimeno L, Semeraro F, Cristofori F, Castellaneta S, Barone M, Indrio F, Gobbetti M, De Angelis M. 2019. Clinical and microbiological effect of a multispecies probiotic supplementation in celiac patients with persistent IBS-type symptoms. J Clin Gastroenterol 53:e117–e125. doi:10.1097/MCG.0000000000001023 29688915 PMC6382041

[40] Giorgi A, Cerrone R, Capobianco D, Filardo S, Mancini P, Zanni F, Fanelli S, Mastromarino P, Mosca L. 2020. A probiotic preparation hydrolyzes gliadin and protects intestinal cells from the toxicity of pro-inflammatory peptides. Nutrients 12:495. doi:10.3390/nu12020495 32075195 PMC7071319

[41] de Almeida NEC, Esteves FG, dos Santos-Pinto JRA, Peres de Paula C, da Cunha AF, Malavazi I, Palma MS, Rodrigues-Filho E. 2020. Digestion of intact gluten proteins by Bifidobacterium species: reduction of cytotoxicity and proinflammatory responses. J Agric Food Chem 68:4485–4492. doi:10.1021/acs.jafc.0c01421 32195585

[42] Zhou J, Li M, Chen Q, Li X, Chen L, Dong Z, Zhu W, Yang Y, Liu Z, Chen Q. 2022. Programmable probiotics modulate inflammation and gut microbiota for inflammatory bowel disease treatment after effective oral delivery. Nat Commun 13:3432. doi:10.1038/s41467-022-31171-0 35701435 PMC9198027

[43] Rizzello CG, De Angelis M, Di Cagno R, Camarca A, Silano M, Losito I, De Vincenzi M, De Bari MD, Palmisano F, Maurano F, Gianfrani C, Gobbetti M. 2007. Highly efficient gluten degradation by lactobacilli and fungal proteases during food processing: new perspectives for celiac disease. Appl Environ Microbiol 73:4499–4507. doi:10.1128/AEM.00260-07 17513580 PMC1932817

[44] Cristofori F, Francavilla R, Capobianco D, Dargenio VN, Filardo S, Mastromarino P. 2020. Bacterial-based strategies to hydrolyze gluten peptides and protect intestinal mucosa. Front Immunol 11:567801. doi:10.3389/fimmu.2020.567801 33224137 PMC7669986

[45] König J, Holster S, Bruins MJ, Brummer RJ. 2017. Randomized clinical trial: effective gluten degradation by Aspergillus niger-derived enzyme in a complex meal setting. Sci Rep 7:13100. doi:10.1038/s41598-017-13587-7 29026170 PMC5638938

[46] Roselli M, Natella F, Zinno P, Guantario B, Canali R, Schifano E, De Angelis M, Nikoloudaki O, Gobbetti M, Perozzi G, Devirgiliis C. 2021. Colonization ability and impact on human gut microbiota of foodborne microbes from traditional or probiotic-added fermented foods: a systematic review. Front Nutr 8. doi:10.3389/fnut.2021.689084 PMC8360115 34395494

[47] Million M, Tomas J, Wagner C, Lelouard H, Raoult D, Gorvel J-P. 2018. New insights in gut microbiota and mucosal immunity of the small intestine. Hum Microbiome J 7–8:23–32. doi:10.1016/j.humic.2018.01.004

[48] Griffiths EA, Duffy LC, Schanbacher FL, Qiao H, Dryja D, Leavens A, Rossman J, Rich G, Dirienzo D, Ogra PL. 2004. In vivo effects of bifidobacteria and lactoferrin on gut endotoxin concentration and mucosal immunity in Balb/c mice. Dig Dis Sci 49:579–589. doi:10.1023/b:ddas.0000026302.92898.ae 15185861

[49] Di Iorio BR, Rocchetti MT, De Angelis M, Cosola C, Marzocco S, Di Micco L, di Bari I, Accetturo M, Vacca M, Gobbetti M, Di Iorio M, Bellasi A, Gesualdo L. 2019. Nutritional therapy modulates intestinal microbiota and reduces serum levels of total and free indoxyl sulfate and p-cresyl sulfate in chronic kidney disease (Medika study). J Clin Med 8:1424. doi:10.3390/jcm8091424 31510015 PMC6780815

[50] Redondo-Useros N, Gheorghe A, Díaz-Prieto LE, Villavisencio B, Marcos A, Nova E. 2019. Associations of probiotic fermented milk (PFM) and yogurt consumption with Bifidobacterium and Lactobacillus components of the gut microbiota in healthy adults. Nutrients 11:651. doi:10.3390/nu11030651 30889821 PMC6470543

[51] Aizawa E, Tsuji H, Asahara T, Takahashi T, Teraishi T, Yoshida S, Ota M, Koga N, Hattori K, Kunugi H. 2016. Possible association of Bifidobacterium and Lactobacillus in the gut microbiota of patients with major depressive disorder. J Affect Disord 202:254–257. doi:10.1016/j.jad.2016.05.038 27288567

[52] Harata G, Kumar H, He F, Miyazawa K, Yoda K, Kawase M, Kubota A, Hiramatsu M, Rautava S, Salminen S. 2017. Probiotics modulate gut microbiota and health status in Japanese cedar pollinosis patients during the pollen season. Eur J Nutr 56:2245–2253. doi:10.1007/s00394-016-1264-3 27412706

[53] Plaza-Díaz J, Fernández-Caballero JÁ, Chueca N, García F, Gómez-Llorente C, Sáez-Lara MJ, Fontana L, Gil Á. 2015. Pyrosequencing analysis reveals changes in intestinal microbiota of healthy adults who received a daily dose of immunomodulatory probiotic strains. Nutrients 7:3999–4015. doi:10.3390/nu7063999 26016655 PMC4488769

[54] Akito K-K, Kensei N, Mai T, Mitsuhisa K, Hiroko K-H, Kazunori S, Hiroshi I, Yusuke G, Kensuke S, Takahiro M, Akira K, Ryoutaro H, Osamu W, Tomoki I, Kouji M, Yuki K, Kazuhito R. 2016. Fermented milk containing Lactobacillus casei strain Shirota preserves the diversity of the gut microbiota and relieves abdominal dysfunction in healthy medical students exposed to academic stress. Appl Environ Microbiol 82:3649–3658. doi:10.1128/AEM.04134-15 27208120 PMC4959178

[55] Pasolli E, De Filippis F, Mauriello IE, Cumbo F, Walsh AM, Leech J, Cotter PD, Segata N, Ercolini D. 2020. Large-scale genome-wide analysis links lactic acid bacteria from food with the gut microbiome. Nat Commun 11:2610. doi:10.1038/s41467-020-16438-8 32451391 PMC7248083

[56] Uriot O, Denis S, Junjua M, Roussel Y, Dary-Mourot A, Blanquet-Diot S. 2017. Streptococcus thermophilus: from yogurt starter to a new promising probiotic candidate? J Funct Foods 37:74–89. doi:10.1016/j.jff.2017.07.038

[57] Liu X, Mao B, Gu J, Wu J, Cui S, Wang G, Zhao J, Zhang H, Chen W. 2021. Blautia—a new functional genus with potential probiotic properties? Gut Microbes 13:1–21. doi:10.1080/19490976.2021.1875796 PMC7872077 33525961

[58] LeBlanc JG, Chain F, Martín R, Bermúdez-Humarán LG, Courau S, Langella P. 2017. Beneficial effects on host energy metabolism of short-chain fatty acids and vitamins produced by commensal and probiotic bacteria. Microb Cell Fact 16:79. doi:10.1186/s12934-017-0691-z 28482838 PMC5423028

[59] Rochet V, Rigottier-Gois L, Sutren M, Krementscki M-N, Andrieux C, Furet J-P, Tailliez P, Levenez F, Mogenet A, Bresson J-L, Méance S, Cayuela C, Leplingard A, Dore J. 2006. Effects of orally administered Lactobacillus casei DN-114 001 on the composition or activities of the dominant faecal microbiota in healthy humans . Br J Nutr 95:421–429. doi:10.1079/BJN20051625 16469162

[60] Caio G, Lungaro L, Segata N, Guarino M, Zoli G, Volta U, De Giorgio R. 2020. Effect of gluten-free diet on gut microbiota composition in patients with celiac disease and non-celiac gluten/wheat sensitivity. Nutrients 12:1832. doi:10.3390/nu12061832 32575561 PMC7353361

[61] De Palma G, Nadal I, Collado MC, Sanz Y. 2009. Effects of a gluten-free diet on gut microbiota and immune function in healthy adult human subjects. Br J Nutr 102:1154–1160. doi:10.1017/S0007114509371767 19445821

[62] Han S, Lu Y, Xie J, Fei Y, Zheng G, Wang Z, Liu J, Lv L, Ling Z, Berglund B, Yao M, Li L. 2021. Probiotic gastrointestinal transit and colonization after oral administration: a long journey. Front Cell Infect Microbiol 11:609722. doi:10.3389/fcimb.2021.609722 33791234 PMC8006270

[63] Pickard JM, Zeng MY, Caruso R, Núñez G. 2017. Gut microbiota: role in pathogen colonization, immune responses, and inflammatory disease. Immunol Rev 279:70–89. doi:10.1111/imr.12567 28856738 PMC5657496

[64] Gibson GR, Hutkins R, Sanders ME, Prescott SL, Reimer RA, Salminen SJ, Scott K, Stanton C, Swanson KS, Cani PD, Verbeke K, Reid G. 2017. Expert consensus document: the international scientific association for probiotics and prebiotics (ISAPP) consensus statement on the definition and scope of prebiotics. Nat Rev Gastroenterol Hepatol 14:491–502. doi:10.1038/nrgastro.2017.75 28611480

[65] Saito Y, Sato T, Nomoto K, Tsuji H. 2018. Identification of phenol- and p-cresol-producing intestinal bacteria by using media supplemented with tyrosine and its metabolites. FEMS Microbiol Ecol 94:fiy125. doi:10.1093/femsec/fiy125 29982420 PMC6424909

[66] Li X, Zhang B, Hu Y, Zhao Y. 2021. New insights into gut-bacteria-derived indole and its derivatives in intestinal and liver diseases. Front. Pharmacol 12. doi:10.3389/fphar.2021.769501 PMC8710772 34966278

[67] Gasaly N, de Vos P, Hermoso MA. 2021. Impact of bacterial metabolites on gut barrier function and host immunity: a focus on bacterial metabolism and its relevance for intestinal inflammation. Front Immunol 12. doi:10.3389/fimmu.2021.658354 PMC8187770 34122415

[68] Dong F, Perdew GH. 2020. The aryl hydrocarbon receptor as a mediator of host-microbiota interplay. Gut Microbes 12:1859812. doi:10.1080/19490976.2020.1859812 33382356 PMC7781536

[69] Agus A, Planchais J, Sokol H. 2018. Gut microbiota regulation of tryptophan metabolism in health and disease. Cell Host Microbe 23:716–724. doi:10.1016/j.chom.2018.05.003 29902437

[70] Lamas B, Hernandez-Galan L, Galipeau HJ, Constante M, Clarizio A, Jury J, Breyner NM, Caminero A, Rueda G, Hayes CL, McCarville JL, Bermudez Brito M, Planchais J, Rolhion N, Murray JA, Langella P, Loonen LMP, Wells JM, Bercik P, Sokol H, Verdu EF. 2020. Aryl hydrocarbon receptor ligand production by the gut microbiota is decreased in celiac disease leading to intestinal inflammation. Sci Transl Med 12:eaba0624. doi:10.1126/scitranslmed.aba0624 33087499

[71] Dinallo V, Marafini I, Di Fusco D, Di Grazia A, Laudisi F, Dwairi R, Paoluzi OA, Monteleone G, Monteleone I. 2019. Protective effects of aryl hydrocarbon receptor signaling in celiac disease mucosa and in poly I:C-induced small intestinal atrophy mouse model. Front Immunol 10:91. doi:10.3389/fimmu.2019.00091 30778350 PMC6369162

[72] Pernomian L, Duarte-Silva M, de Barros Cardoso CR. 2020. The aryl hydrocarbon receptor (AHR) as a potential target for the control of intestinal inflammation: insights from an immune and bacteria sensor receptor. Clin Rev Allergy Immunol 59:382–390. doi:10.1007/s12016-020-08789-3 32279195

[73] Vanholder R, Nigam SK, Burtey S, Glorieux G. 2022. What if not all metabolites from the uremic toxin generating pathways are toxic? A hypothesis. Toxins 14:221. doi:10.3390/toxins14030221 35324718 PMC8953523

[74] Wang X, Gibson GR, Costabile A, Sailer M, Theis S, Rastall RA. 2019. Prebiotic supplementation of in vitro fecal fermentations inhibits proteolysis by gut bacteria, and host diet shapes gut bacterial metabolism and response to intervention. Appl Environ Microbiol 85:e02749-18. doi:10.1128/AEM.02749-18 30824442 PMC6495761

[75] Nowak A, Libudzisz Z. 2006. Influence of phenol, p-cresol and indole on growth and survival of intestinal lactic acid bacteria. Anaerobe 12:80–84. doi:10.1016/j.anaerobe.2005.10.003 16701619

[76] Elgaali H, Hamilton-Kemp TR, Newman MC, Collins RW, Yu K, Archbold DD. 2002. Comparison of long-chain alcohols and other volatile compounds emitted from food-borne and related Gram positive and Gram negative bacteria. J Basic Microbiol 42:373–380. doi:10.1002/1521-4028(200212)42:6<373::AID-JOBM373>3.0.CO;2-4 12442299

[77] Celiachia AI. 2020. Registro nazionale sezione 2: alimenti senza glutine. https://www.salute.gov.it/portale/temi/documenti/integratori/Reg_naz_sezione_alimenti_senza_glutine_per_prodotto.pdf.

[78] Andrews S, Krueger F, Segonds-Pichon A, Biggins L, Krueger C, Wingett S. 2010. FastQC. a qual control tool high throughput seq data 370.

[79] Ewels P, Magnusson M, Lundin S, Käller M. 2016. MultiQC: summarize analysis results for multiple tools and samples in a single report. Bioinformatics 32:3047–3048. doi:10.1093/bioinformatics/btw354 27312411 PMC5039924

[80] Bolyen E, Rideout JR, Dillon MR, Bokulich NA, Abnet CC, Al-Ghalith GA, Alexander H, Alm EJ, Arumugam M, Asnicar F, et al.. 2019. Reproducible, interactive, scalable and extensible microbiome data science using QIIME 2. Nat Biotechnol 37:852–857. doi:10.1038/s41587-019-0209-9 31341288 PMC7015180

[81] Callahan BJ, McMurdie PJ, Rosen MJ, Han AW, Johnson AJA, Holmes SP. 2016. DADA2: high-resolution sample inference from Illumina amplicon data. Nat Methods 13:581–583. doi:10.1038/nmeth.3869 27214047 PMC4927377

[82] De Angelis M, Ferrocino I, Calabrese FM, De Filippis F, Cavallo N, Siragusa S, Rampelli S, Di Cagno R, Rantsiou K, Vannini L, Pellegrini N, Lazzi C, Turroni S, Lorusso N, Ventura M, Chieppa M, Neviani E, Brigidi P, O’Toole PW, Ercolini D, Gobbetti M, Cocolin L. 2020. Diet influences the functions of the human intestinal microbiome. Sci Rep 10:4247. doi:10.1038/s41598-020-61192-y 32144387 PMC7060259

[83] Portincasa P, Bonfrate L, Vacca M, De Angelis M, Farella I, Lanza E, Khalil M, Wang DQ-H, Sperandio M, Di Ciaula A. 2022. Gut microbiota and short chain fatty acids: implications in glucose homeostasis. Int J Mol Sci 23:1105. doi:10.3390/ijms23031105 35163038 PMC8835596

[84] Hsu BB, Gibson TE, Yeliseyev V, Liu Q, Lyon L, Bry L, Silver PA, Gerber GK. 2019. Dynamic modulation of the gut microbiota and metabolome by bacteriophages in a mouse model. Cell Host Microbe 25:803–814. doi:10.1016/j.chom.2019.05.001 31175044 PMC6579560

[85] McMurdie PJ, Holmes S. 2013. phyloseq: an R package for reproducible interactive analysis and graphics of microbiome census data. PLoS One 8:e61217. doi:10.1371/journal.pone.0061217 23630581 PMC3632530

[86] Oksanen J, Blanchet FG, Kindt R, Legendre P, Minchin P, O’Hara RB, Simpson G, Solymos P, Stevens MHH, Wagner H. 2013. Vegan: community ecology package. Package version. 2.0-10. CRAN.

[87] Wickham H, Chang W, Wickham MH. 2016. Package ‘ggplot2.’ Creat elegant data vis using gramm graph version 2 p 1–189

[88] Segata N, Izard J, Waldron L, Gevers D, Miropolsky L, Garrett WS, Huttenhower C. 2011. Metagenomic biomarker discovery and explanation. Genome Biol 12:R60–R60. doi:10.1186/gb-2011-12-6-r60 21702898 PMC3218848

