# Supplementary material for: Novel probiotic preparation with in vivo gluten-degrading activity and potential modulatory effects on the gut microbiota
Source: Microbiol Spectr. 2024 Jun 11;12(7):e03524-23. doi: 10.1128/spectrum.03524-23 (PMC11218521; doi:10.1128/spectrum.03524-23)
Supplement: Supplemental figures — Fig. S1-S3. [file spectrum.03524-23-s0001.pdf]

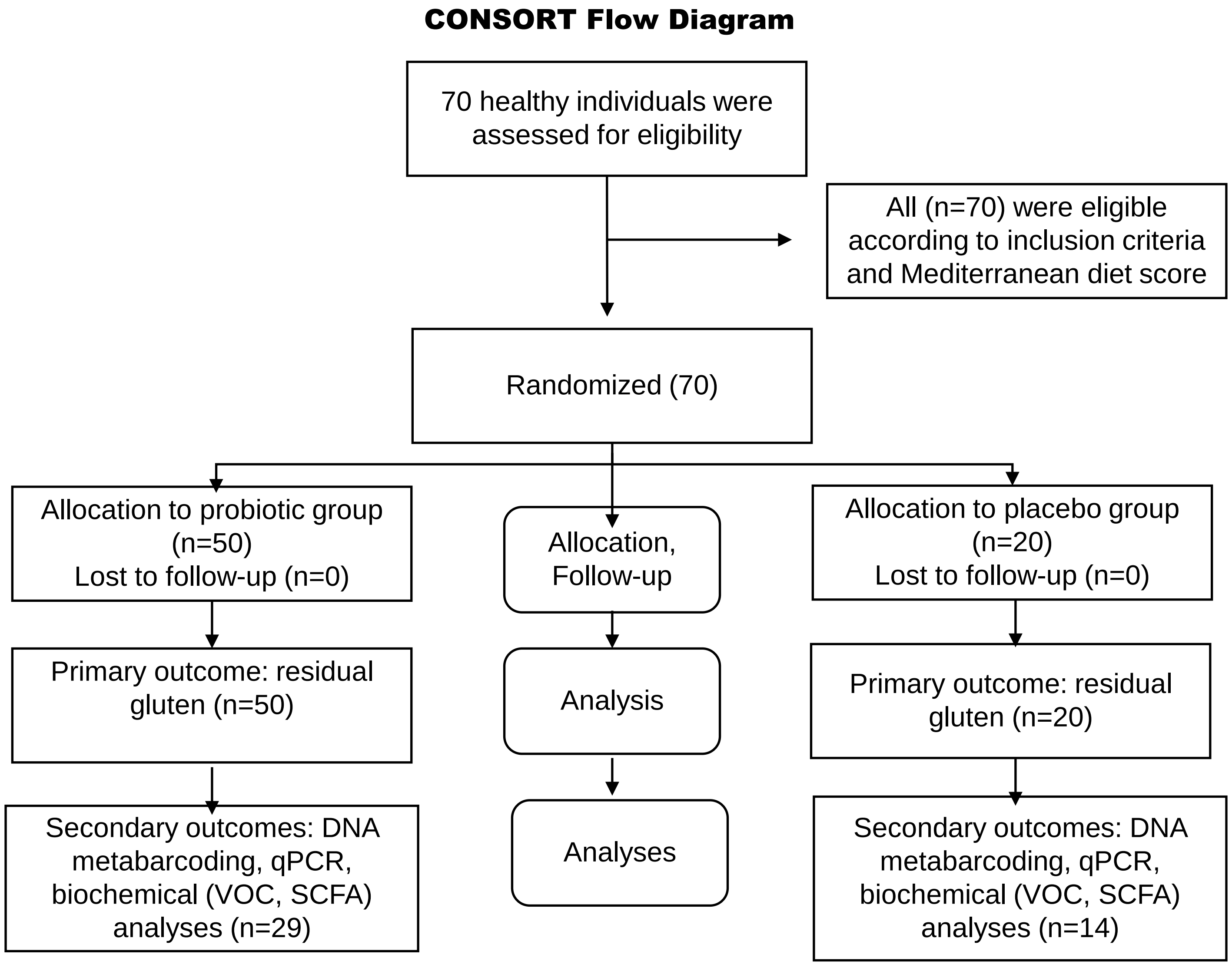

Figure S1. Consort flow Diagram of the intervention study

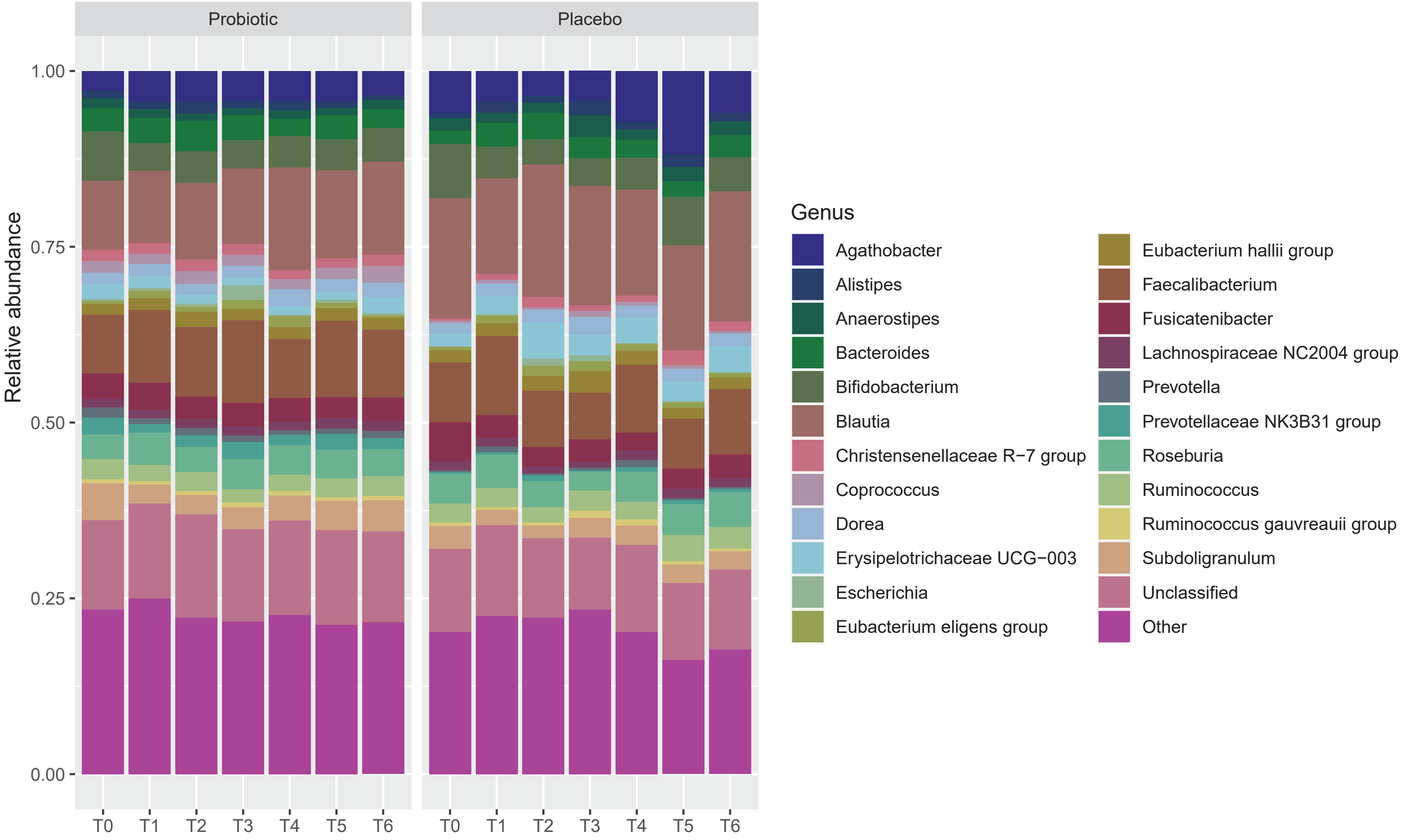

Figure S2. Relative abundances of the top 30 taxa. Top 30 genera in samples with different treatment (probiotic vs placebo) across sampling timepoints: baseline (T0); 10 days of GFD (T1); 4 days of 50 mg/day gluten intake (T2); 4 days of 1 g/day gluten intake (T3); 4 days of 3 g/day gluten intake (T4); 20 days of 10g/day gluten intake (T5), of which 10 last days were the wash-out (T6). Unclassified are taxa in the top 30 which are not classified to genus level. “Other” signifies taxa not in the top 30. OTU were agglomerated on genus level and genera with abundances <0.02% were removed.

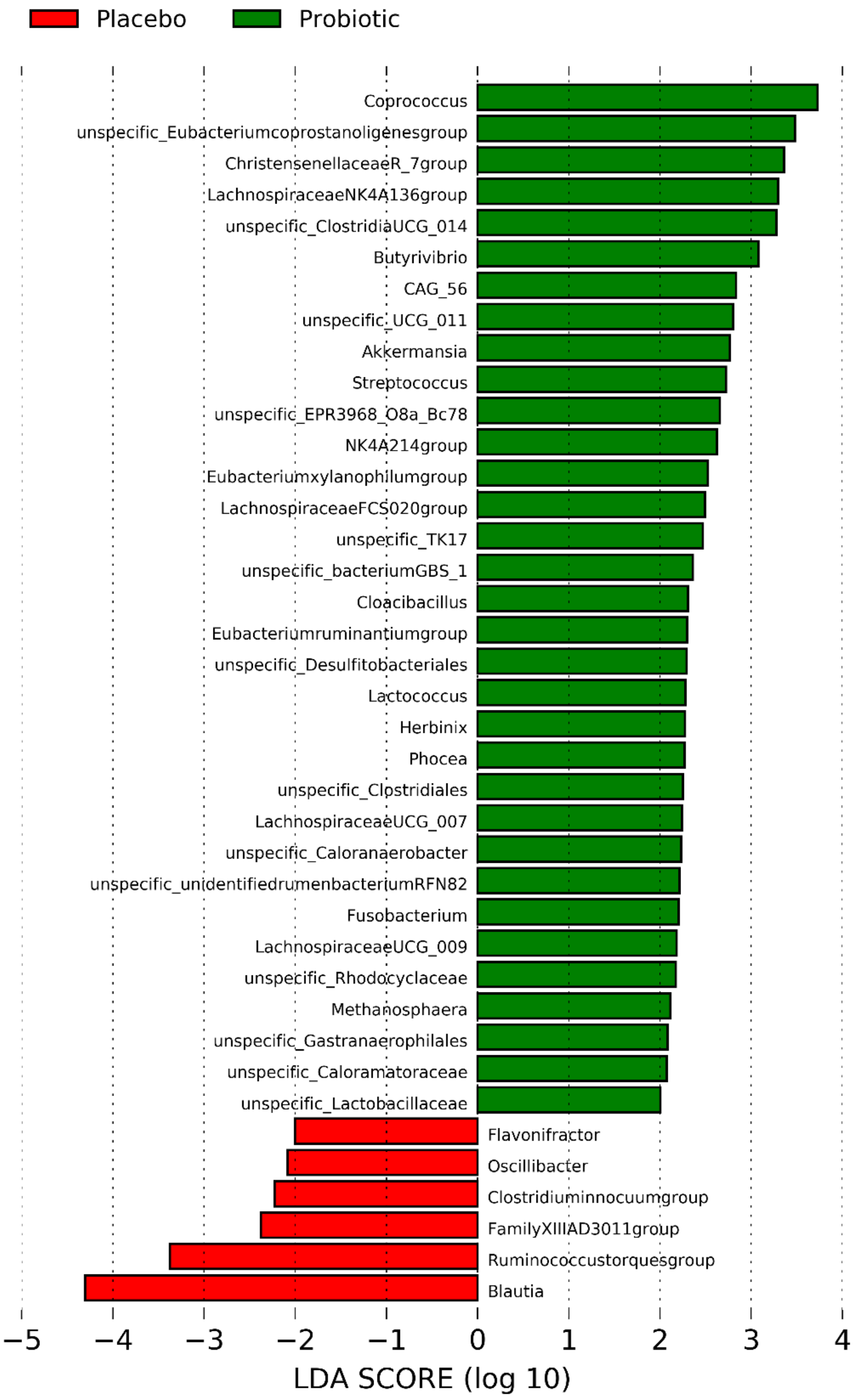

Figure S3. Differential abundance species found between placebo and probiotic group as calculated using Lefse algorithm. The probiotic taxa are indicated with a positive LDA score (green), and the placebo taxa have a negative score (red). Time points refer to 10 days of GFD (T1); 20 days of 10g/day gluten intake (T5) of which 10 last days were the wash-out. (T6).
