# Supplementary material for: Novel probiotic preparation with in vivo gluten-degrading activity and potential modulatory effects on the gut microbiota
Source: Microbiol Spectr. 2024 Jun 11;12(7):e03524-23. doi: 10.1128/spectrum.03524-23 (PMC11218521; doi:10.1128/spectrum.03524-23)
Supplement: Supplemental guidelines diet — Guidelines for a correct gluten free diet. [file spectrum.03524-23-s0002.pdf]

Guidelines for a correct gluten free diet

In vivo challange

October - November - December 2021

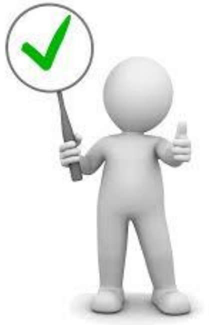

What you can eat

1- Naturally gluten-free (and unprocessed) **foods**.

- Vegetable origin: rice, corn, sorghum, millet, teff, quinoa, buckwheat, fruits and vegetables.
- Animal origin: meat, fish, eggs.

2- Gluten Free or “Senza Glutine” word.

- Processed food that the manufacturer guarantees suitable. May include additional words on the label (“specifically formulated for people intolerant to gluten”/ “specifically formulated for coeliacs”/ “suitable for people intolerant to gluten” “suitable for coeliacs”).

3- The gluten-free symbol (an ear of corn crossed out in a circle)

- Is a registered trademark (FACULTATIVE). Naturally “gluten-free" products (1) have not this symbol.

!!! If the ingredients of an unsafe food do not include gluten or cereals containing gluten (wheat, rye, oats, barley, spelt, kamut® and derived products), it is NOT automatically considered suitable. The label 'gluten-free' is a guarantee . Without this wording the product could potentially be unsuitable, so it is recommended not to consume it.

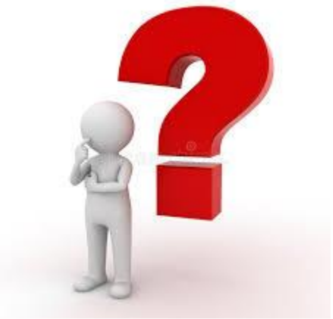

What you have to check

1. Labelling is mandatory when **allergens** are used as an **ingredient** in the product (packed products and restaurant).
2. The absence of the wording « **May contain traces of gluten** » does not give a guarantee such as the claim «**gluten-free**».

Reg. (EU) 1169/2011
Allergens: Allergy-causing or intolerant substances (such as wheat derivatives and cereals containing gluten..) should be highlighted more clearly in the list of ingredients using graphic devices (e.g., **bold**, color or underlining).

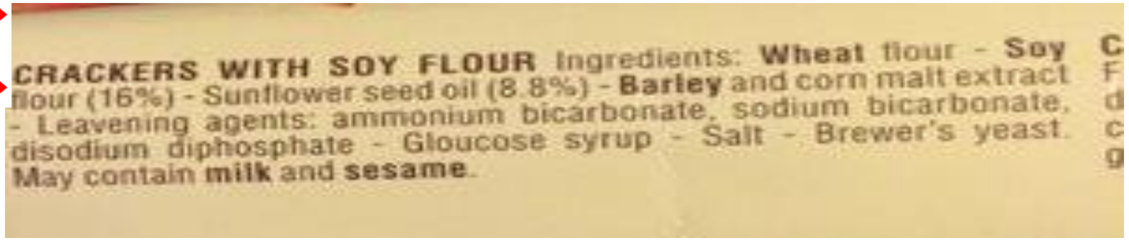

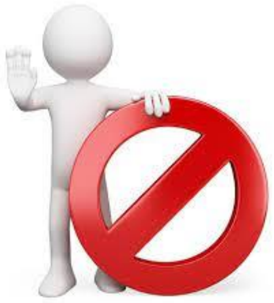

Cereal containing GLUTEN

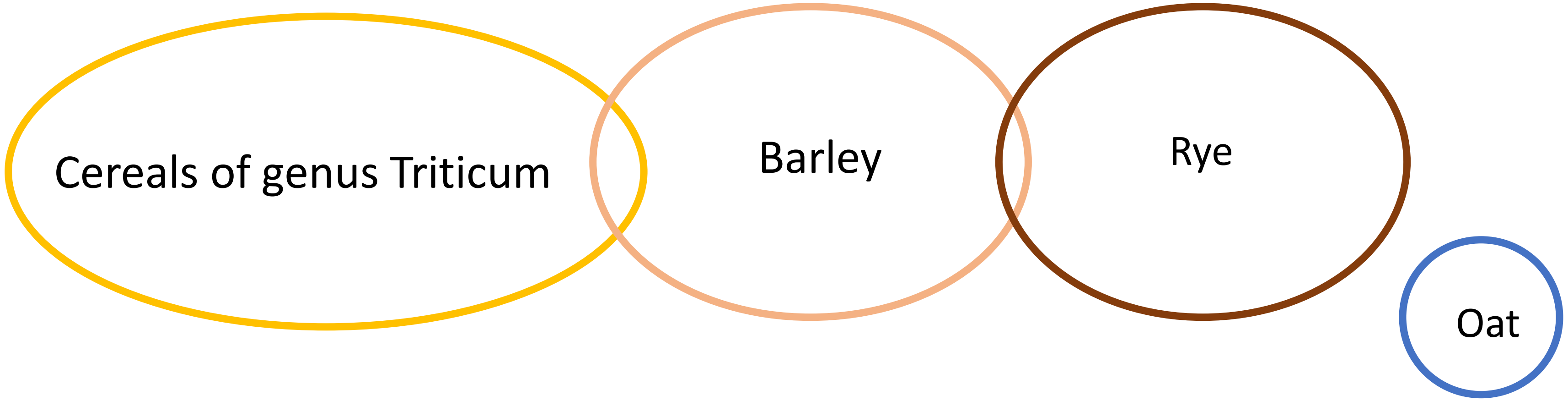

CEREALS OF GENUS TRITICUM (e.g.)

- Common wheat - triticum aestivum;
- Durum wheat - triticum durum;
- Khorasan wheat - triticum turanicum;
- Spelt or large spelt - triticum spelta;
- Spelt or medium spelt - triticum dicoccum;
- Monococcus or small spelt - triticum monococcum

Sustainable Mediterranean Diet

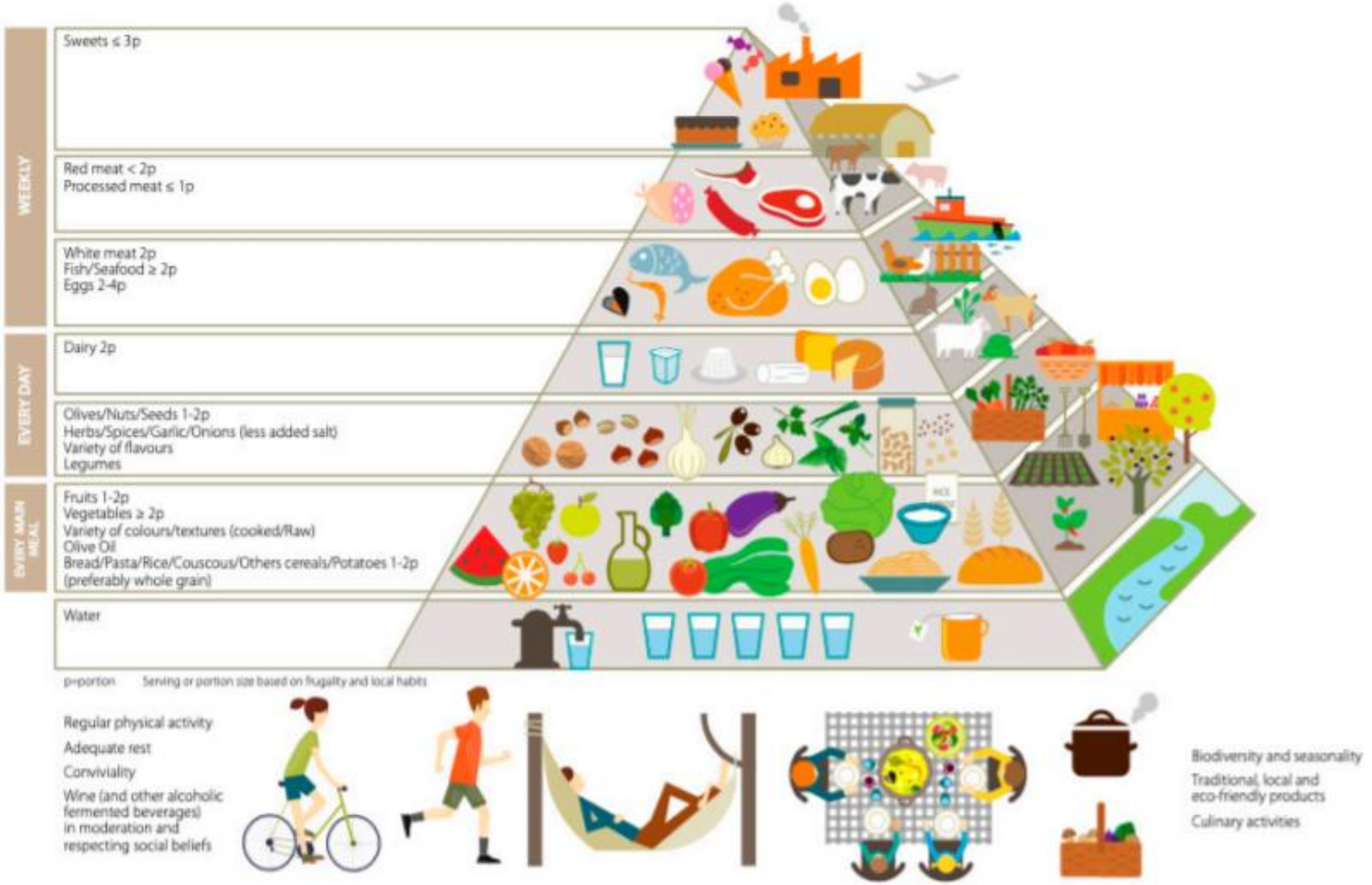

Serra-Majem, L., Tomaino, L., Dernini, S., Berry, E. M., Lairon, D., *et al.* (2020) 'Updating the mediterranean diet pyramid towards sustainability: Focus on environmental concerns', *International Journal of Environmental Research and Public Health*. doi: 10.3390/ijerph17238758.

Food groups

AiC Associazione Italiana Celiachia

1- CEREALS AND TUBERS? 3-5 portions a day in this group.

2- FRUIT AND VEGETABLES? 3-5 portions a day in this group.

3- MILK AND DAIRY PRODUCTS? 2-3 portions a day in this group.

4- MEAT FISH AND EGGS? 1 - 2 portions a day in this group (alternate weekly frequencies).

5- FATS FOR SEASONING? 3-4 portions a day olive oil.

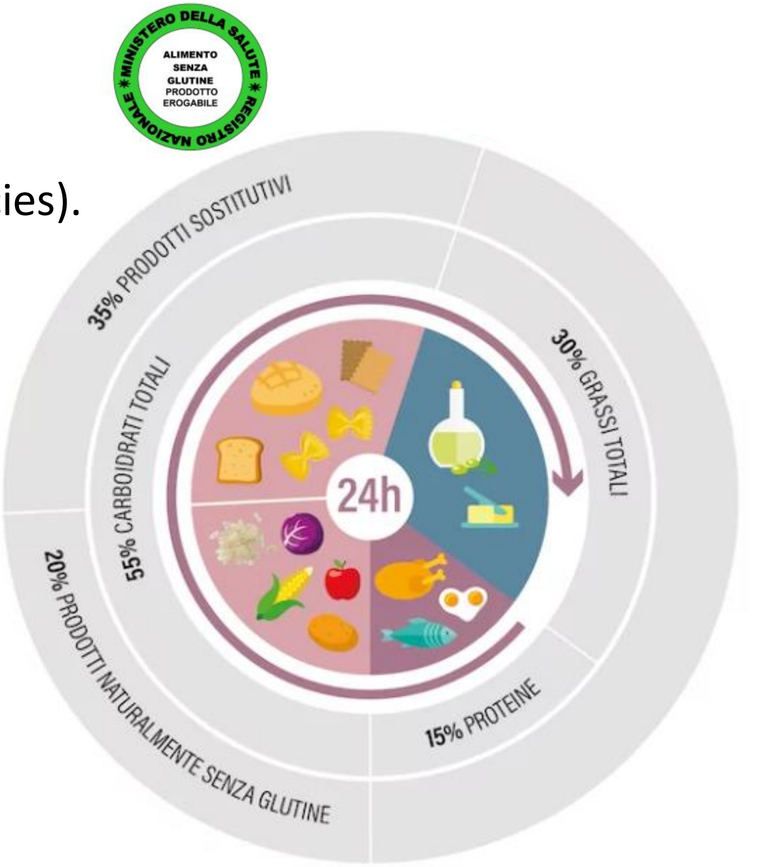

AIC: Associazione Italiana celiachia. Image modified.

Grains and flours

Gluten free

- Amaranth
- Buckwheat
- Chestnut
- Corn (maize)
- Millet
- Quinoa
- Rice
- Sorghum
- Teff
- Gluten free oats

Not gluten free

- Barley
- Bulgar wheat
- Couscous
- Spelled
- Durum wheat
- Freekeh
- Kamut
- Pearl barley
- Rye
- Semolina
- Spelt
- Triticale
- Wheat
- Porridge oats
- Oat milk
- Oat snacks not labelled gluten free

Need to check

- Polenta (cornmeal)
- Soya
- Flours of cereal allowed
- Tapioca

Meat and poultry

Gluten free

- All fresh meats and poultry
- Plain cooked meats
- Smoked meats

Not gluten free

- Meat and poultry cooked in batter or breadcrumbs
- Breaded ham

Meatless alternatives

Need to check

- Marinated tofu
- Soya mince
- Falafel
- Vegetable and vegan burgers/sausages
- Plain tofu

Fish and shellfish

Gluten free

- All dried, fresh, and smoked fish
- Shellfish
- Fish canned in brine, oil and water

Not gluten free

- Fish or shellfish in batter or breadcrumbs not labelled gluten free
- Fish cakes and fish fingers not labelled gluten free

Need to check

- Fish pastes
- Fish patés
- Fish in sauce

Milk and derivatives

Gluten free

- Fresh milk
- Fresh and long-life cream
- Buttermilk
- Plain yoghurt

Not gluten free

- Yoghurt with muesli or wholegrains

Need to check

- Coffee and tea whiteners
- Condensed milk
- Dried milk
- Fruit and flavored yoghurt
- Soya desserts
- Soya milk
- Soya yoghurt
- Rice milk
- Nut milks
- Cheese
- Seasoned cream
- Whipped, spray, vegetable cream

Fats, oils, eggs

Gluten free

- Butter
- Cooking oils
- Ghee
- Lard
- Reduced and low fat spreads
- Eggs

Need to check

- Margarine

Fruits and vegetables

Gluten free

- All canned, dried, fresh, frozen and juiced pure fruits and vegetables
- Pickled vegetables in spirit vinegar

Not gluten free

- Vegetables and fruit in batter, breadcrumbs or dusted with flour

Need to check

- Fruit pie fillings
- Processed vegetable products
- Vegetables pickled in barley malt vinegar

Nuts seeds and pulses

Gluten free

- Plain nuts and seeds
- All pulses (peas, beans, lentils)

Need to check

- Dry roasted nuts
- Pulses in flavoured sauce (such as baked beans)

Spreads, fillings and dips

Gluten free

- Conserves
- Glucose syrup
- Golden syrup
- Honey
- Jam
- Marmalade
- Molasses
- Yeast extract

Need to check

- Lemon curd
- Mincemeat
- Peanut and other nut butter
- Prepared dips

Soups, sauces, pickles and seasonings

Gluten free

- Vinegars
- Garlic puree
- Ground pepper
- Mint sauce
- Individual herbs and spices
- Mixed herbs and spices
- Salt
- Tomato puree

Need to check

- Barley malt vinegar
- Blended and powdered seasonings
- Canned, packet or fresh soups
- Chutney
- Curry powder
- Dressings
- Mayonnaise
- Mustard products
- Packed and jarred sauces
- Salad cream
- Tamari (japanese soy sauce)
- Tomato sauce
- Mustard powder
- Chinese soy sauce

Home baking

Gluten free

- Arrowroot
- Bicarbonate of soda
- Corn starch (flour)
- Cream of tartar
- Potato starch (flour)
- Fresh and dried yeast
- Glacé cherries

Need to check

- Baking powder
- Cake decorations
- Marzipan
- Ready to use icings
- Artificial sweeteners
- Food colouring
- Gelatine
- Ground almonds
- Icing sugar

Not gluten free

- Batter mixes
- Breadcrumbs
- Stuffing mix

Drinks and alcohol

Gluten free

- Coffee
- Fruit juice
- Squash
- Tea
- Water
- Gluten free beers and lagers
- Port
- Sherry
- Spirits
- Wine

Need to check

- Cocoa
- Cider
- Liqueurs

Not gluten free

Beer

Stanford Children's Health | Lucile Packard Children's Hospital Stanford

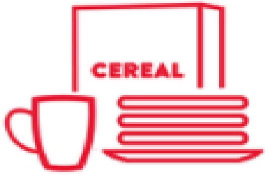

Gluten-free meals ideas: Breakfast

- Rice cereals or corn cereals or other gluten free cereals, with milk, nut milks, fresh fruit
- Corn tortillas, scrambled eggs, chopped tomatoes, melted cheese
- Cream of rice cereal with chopped almonds and milk
- Omelette with onions, peppers, and tomatoes, with soft corn tortillas
- Gluten-free pancakes
- Gluten-free yogurt with fruits
- Hard-boiled eggs

Stanford Children's Health | Lucile Packard Children's Hospital Stanford

Gluten-free meals ideas: Lunch

- Sliced turkey with lettuce, tomato, and mayonnaise on warmed corn tortillas with baby carrots
- Grilled sliced chicken over mixed greens, with red peppers, sliced tomatoes, broccoli florets, and chickpeas, served with oil and vinegar or gluten-free salad dressing
- Toasted gluten-free bread or warmed corn tortillas, with tuna fish made with mayonnaise, chopped onion, sliced tomato, shredded lettuce, and chopped cucumber
- Grilled salmon or tuna served over mixed greens with shredded carrots, chopped tomatoes, and cucumbers. Serve with oil and vinegar, or gluten-free salad dressing, gluten-free rice crackers, and lemon wedges
- Grilled chicken, salmon, or tuna, with shredded lettuce, sliced tomatoes, baby carrots, and gluten-free rice cakes

Gluten-free meals ideas: Lunch

- Gluten-free ham on gluten-free toast or warmed corn tortillas with mustard and coleslaw
- Cottage cheese with mixed fruit
- Grilled chicken cutlet marinated in garlic, oil, and lemon, served over chopped romaine lettuce, with gluten-free Caesar dressing, parmesan cheese, and gluten-free rice crackers
- Grilled or broiled sirloin burger with lettuce, tomato, sliced onion, ketchup, and a mixed salad with oil and gluten-free vinegar
- Grilled chicken marinated in garlic, oregano, oil, salt, and pepper, with a sweet potato, butter, and mixed veggies
- Chicken salad made with cooked chicken, mayo, onions, walnuts, and grapes, over a mixed green salad

Stanford Children's Health | Lucile Packard Children's Hospital Stanford

Gluten-free meals ideas: Dinner

- Salmon baked with mustard, and honey, served with brown rice and steamed green beans
- Hard-boiled egg, sliced, with steamed green beans, baby spinach, sliced cucumber, sliced tomato, and chickpeas with oil and vinegar or gluten-free salad dressing
- Grilled chicken cutlet marinated in garlic, oil, and onion powder, served with cooked brown rice, steamed broccoli, and mixed greens served with oil and vinegar or gluten-free salad dressing
- Broiled skirt steak with garlic, onion powder, and a dash of salt, served with steamed cauliflower and a medium baked potato with butter or margarine

Gluten-free meals ideas: Dinner

Stanford Children's Health | Lucile Packard Children's Hospital Stanford

- Baked flounder cooked with chopped onions, tomatoes, cilantro, garlic, and onion powder, served with steamed spinach, rice, and a mixed green salad sliced tomato and cucumber and oil and vinegar or gluten-free salad dressing
- Pork loin cut into two-inch cubes of pineapple and cherry tomatoes marinated in gluten-free Italian dressing, grilled, and served with steamed broccoli and corn with butter or margarine and a dash of salt
- Roasted chicken with carrots, potatoes, and onions, seasoned with garlic, onion powder, salt, pepper, and Italian herbs
- Grilled or baked chicken, shrimp, or veal placed in a casserole dish and topped with tomato sauce, mozzarella, and parmesan cheese, served with gluten-free pasta

Gluten-free meals ideas: Dinner

- Rice, corn, or quinoa pasta with tomato sauce and a mixed green salad with favorite gluten-free dressing
- Grilled shrimp over a mixed green salad with baby potatoes and favorite gluten-free dressing
- Hand-pressed hamburger or turkey burger (100% pure ground beef or turkey), with onion and sliced tomato, baked sweet potato fries, and green beans
- Gluten-free pizza baked and served with mixed green salad and gluten-free salad dressing
- Pork loin cut into two-inch cubes of pineapple and cherry tomatoes marinated in gluten-free Italian dressing, grilled, and served with steamed broccoli and corn with butter or margarine and a dash of salt

Stanford Children's Health | Lucile Packard Children's Hospital Stanford

Gluten-free meals ideas: Snacks

- Fresh fruit
- Cheese*
- Rice crackers with peanut butter or cheese*
- Popcorn*
- Sliced veggies with gluten-free bean dip*
- Canned fruit in its own juices

- Plain yoghurt
- Applesauce with cinnamon
- Baby carrots and snow peas with hummus*
- String cheese and dried fruit*
- Pudding*
- Rice cakes*
- Nuts with dried fruit*

- Strawberries with Cool Whip
- Plain peanuts or almonds*
- Rice cakes with cream cheese and jam*
- Vanilla ice cream, sorbets, ice milk*
- Hard-boiled egg

** Double-check labels particularly in these items.*

ALLOWED FOODS

- ✓ Rice grains
- ✓ Corn (maize) kernels, steamed
- ✓ Buckwheat grains
- ✓ Amaranth grains
- ✓ Millet seed
- ✓ Quinoa seed
- ✓ Sorghum grains
- ✓ Teff grains
- ✓ Fonio grains
- ✓ Tubers (potato, sweet potato, Mexican potato, cassava, Jerusalem artichoke, etc.).
- ✓ Substitute products in the National Register of the Ministry of Health * and other Gluten Free substitute foods

UNSAFE FOODS (TO CHECK)

- Mixes of permitted cereals, mixes of permitted cereals and pulses
- Flour, starch, starches (e.g. cornstarch), groats, meal, creams and flakes of permitted cereals
- Flour for precooked and instant polenta, ready-made polenta
- Flour from: chickpeas, soya, chestnuts, almonds, hazelnuts, etc.
- Malt, malt extract from permitted cereals
- Malt extract from prohibited cereals
- Tapioca (manioc flour)
- Deglutinated wheat starch
- Breakfast products made from permitted cereals (puffed, flaked, muesli)
- Waffles, galettes of permitted cereals
- Bran of permitted cereals
- Vegetable and dietary fibers
- Packaged popcorn
- Ready-made risottos (in sachets, frozen, flavoured)
- Couscous, tacos, tortillas from permitted cereals
- Replacement products (e.g. flour mixes, bread and bread substitutes, pasta)
- Oat-based products (flours, pasta, biscuits, etc.)

`*REGISTRO NAZIONALE SEZIONE 2: Alimenti senza glutine
https://www.salute.gov.it/portale/temi/documenti/integratori/Reg_naz_sezione_alimenti_senza_glutine_per_prodotto.pdf

FORBIDDEN FOODS

- Wheat
- Spelt
- Barley
- Rye
- Einkorn (monococcus)
- Khorasan wheat (usually marketed as Kamut®)
- Spelt
- Triticale
- Grain oats (intended for the final consumer)
- Flour, starches, groats, meal, creams and flakes of prohibited cereals
- First courses prepared with prohibited cereals (pasta, stuffed pasta, potato gnocchi, gnocchi alla romana, pizzoccheri, crepes)
- Bread and bakery substitutes, sweet and savoury, prepared with prohibited cereals (breadcrumbs, breadcrumbs, focaccia, pizza, piadine, panzerotti, breadsticks, crackers, rusks, taralli, crostini, salatini, cracotte, biscuits, snacks, pastries, cakes)
- Wheat germ
- Ethnic flours and derivatives: bulgur (boulgour or burghul), couscous (from prohibited cereals), cracked wheat, frik, greis, greunkern, seitan, tabulè
- Bran from prohibited cereals
- Malt from prohibited cereals
- Breakfast products made from prohibited cereals (puffed, flaked, muesli, porridge)
- Polenta taragna (if buckwheat flour is mixed with wheat flour)

ALLOWED FOODS

- ✓ All types of meat, fish, mollusks and crustaceans as they are (fresh or frozen) not mixed with other ingredients (excluding salt, sugar, sulphites, citric acid, ascorbic acid, sodium citrate, sodium ascorbate and sodium carbonates).
- ✓ Preserved fish: au naturel, in oil, in vinegar, in salt, smoked, free of additives, flavourings and other substances (excluding sulphites, citric acid and ascorbic acid)
- ✓ Eggs
- ✓ Liquid pasteurized eggs (whole, yolks or whites) free of additives, flavourings and other substances (unflavoured)
- ✓ Bresaola, Culatello, Lardo, Prosciutto crudo, Speck

UNSAFE FOODS (TO CHECK)

- Cold cuts and sausages made from pork, beef or poultry (coppa, cotechino, mortadella, pancetta, ham, salami, sausage, frankfurters, pig's trotter, chicken or turkey, etc.).
- Preserved meat (e.g. canned meat, meat in jelly)
- Hamburgers
- Preserved fish: in brine, in oil, in vinegar, in salt, smoked, with other added substances (excluding sulphites, citric acid and ascorbic acid)
- Homogenized meat, fish, ham
- Ready-made or precooked meat or fish dishes
- Flavoured pasteurised liquid eggs (whole, yolks or whites)
- Eggs (whole, yolks or whites) powdered
- Surimi

FORBIDDEN FOODS

- Breaded meat or fish (cutlets, sticks, fried fish, etc.) or floured or mixed with breadcrumbs (hamburgers, meatballs, etc.) or cooked in sauces and sauces thickened with prohibited flours

3. MILK, DAIRY PRODUCTS, CHEESE AND VEGETABLE SUBSTITUTES

ALLOWED FOODS

- ✓ Milk: fresh (pasteurised), long-life (UHT, sterilised), lactose-free or highly digestible, not containing added flavourings or other substances (except vitamins and/or minerals).
- ✓ Powdered milk with no other added ingredients
- ✓ Milk for early childhood (0-12 months)
- ✓ Fermented milk, probiotics (containing only milk/yoghurt, sugar and milk enzymes)
- ✓ Fresh and ripened cheeses, including delactose and/or light cheeses
- ✓ Grated Parmigiano Reggiano DOP and Grana Padano DOP cheeses
- ✓ Natural yoghurt (low-fat or whole) even if lactose-free
- ✓ Creamy white yoghurt without added thickeners, flavourings or other substances (containing only yoghurt, sugar and lactic ferments)
- ✓ Greek yoghurt (containing only milk, cream and lactic ferments) without added flavourings or other substances
- ✓ Cream: fresh (pasteurised) and long-life (UHT) even if lactose-free, not mixed with other ingredients, excluding carrageenan (E 407)

UNSAFE FOODS (TO CHECK)

- Cream: long-life (UHT) seasoned (mushroom, salmon, etc.), whipped, sprayed, vegetable
- Yoghurt with fruit, 'flavoured with...', creamy
- Creamy white yoghurt with added thickeners, flavours or other substances
- Greek yoghurt with added thickening agents, flavours or other substances
- Soya yoghurt, rice
- Processed cheese, sliced processed cheese, vegetable (e.g. tofu)
- Light cheese with added thickeners, flavourings or other substances
- Spreadable cheese with added thickeners, flavourings or other substances
- Flaked milk with added thickening agents, flavourings or other substances
- Grated cheese or mix of grated cheeses excluding Grana Padano DOP and Parmigiano Reggiano DOP
- Creams, puddings, desserts, panna cotta based on milk, soya, rice
- Milk powder with added ingredients
- Condensed milk
- Milk with added/enriched fibres, cocoa, flavourings or other substances (except vitamins and/or minerals)
- Growth milk (1-3 years)
- Fermented milk, probiotics with added thickeners, flavourings or other substances
- Beverages based on milk, soy, rice, almonds, coconut, etc.
- Oat-based drinks
- Homogenized cheese

3. MILK, DAIRY PRODUCTS, CHEESE AND VEGETABLE SUBSTITUTES

FORBIDDEN FOODS

- Ready-made cheese dishes breaded with prohibited flours
- Malt, cereal and biscuit yoghurt
- Cereal or biscuit milk

ALLOWED FOODS

- ✓ All types of vegetables in their natural state (fresh, dried, frozen, deep-frozen, freeze-dried).
- ✓ Vegetables, preserved mushrooms (in brine, vinegar, oil, salt) if they consist solely of: vegetables and/or mushrooms, water, salt, oil, vinegar, sugar, sulphites, ascorbic acid, citric acid, spices and herbs.
- ✓ Steamed/blanched vegetables, whether or not with salt, ascorbic acid and citric acid added.
- ✓ Fresh, dried, frozen mushrooms as is
- ✓ All pulses as they are (fresh, frozen, dried and canned) or consisting only of water, salt, sugar, sulphur dioxide, ascorbic acid, citric acid, tomato paste: carobs, chickpeas, chickling vetch, beans, broad beans, lentils, lupins, peas, soybeans
- ✓ Minestrone preparations (frozen, fresh, dried) consisting solely of vegetables (vegetables, legumes, potatoes)

UNSAFE FOODS (TO CHECK)

- Minestrone preparations consisting of vegetables and other ingredients
- Vegetable purees
- Soups and broths with permitted cereals
- Pulses, pulses and cereals mixes permitted
- Pre-cooked frozen vegetable dishes (e.g. vegetables and cheese)
- Pre-cooked frozen potatoes
- Potato chips packed in bags (snacks)
- Ready-made, instant or frozen mashed potatoes
- Potato flakes
- Preserved, steamed/blanched vegetables mixed with other ingredients
- Grilled vegetables (in brine, in oil, frozen)
- Homogenized vegetables

FORBIDDEN FOODS

- Vegetables (minestrone, soups, etc.) with prohibited cereals
- Breaded, floured, battered vegetables with prohibited ingredients

ALLOWED FOODS

- ✓ All types of fruit (fresh and frozen)
- ✓ All types of nuts with and without shell (roasted, salted, even if with added vegetable oils)
- ✓ Dehydrated fruit, dried and not floured even if sugar, glucose syrup or glucose-fructose syrup, honey, vegetable oils, sulphites, citric acid and ascorbic acid have been added (dates, figs, prunes, sultanas, etc.)
- ✓ Fruit in syrup, whether or not citric acid, ascorbic acid, juice, sugar, glucose syrup or glucose-fructose syrup has been added.
- ✓ Smoothies, mousses, and fruit purées consisting solely of fruit, sugar, ascorbic acid (E300 or vitamin C) and citric acid (E330)

UNSAFE FOODS (TO CHECK)

- Dehydrated, dried fruit not coated in flour with other ingredients
- Candied, caramelised, glazed fruit
- Smoothies, mousses and fruit purées mixed with other ingredients
- Homogenized fruit
- Flour and dried fruit grains (coconut, almonds, hazelnuts, chestnuts, etc.)

FORBIDDEN FOODS

- Floured dehydrated fruit (dried figs, etc.)

ALLOWED FOODS

- ✓ Carbonated/sparkling (still) soft drinks such as soda, tonic water, cola, chinotto, orangeade, etc., including low-calorie or zero-calorie variants (e.g. "light" and "zero")
- ✓ Fruit nectars, fruit juices and fruit drinks not containing added vitamins or other substances (preservatives, flavourings, colourings, etc.), except: ascorbic acid (E300 or vitamin C), citric acid (E330), sugar, fructose, glucose or glucose-fructose syrup

UNSAFE FOODS (TO CHECK)

- Beverages based on milk, soy, rice, almonds, coconut, etc...
- Oat-based drinks
- Ginseng coffee-flavoured drinks
- Milkshakes (ready-made mixes, in powder form)
- Saline supplements (liquid / powder)
- Flavoured water with added minerals and/or vitamins
- Nectars, fruit juices and drinks with added vitamins or other substances
- Preparations for chocolate/cocoa, cappuccino drinks
- Syrups for soft drinks and slushies
- Effervescents for beverages

FORBIDDEN FOODS

ALLOWED FOODS

- ✓ Coffee, decaffeinated coffee, coffee pods and capsules
- ✓ Tea, decaffeinated tea, camomile, herbal teas (loose, filter, sachet) consisting of herbs, fruits and essential oils and flavourings

UNSAFE FOODS (TO CHECK)

- Ginseng coffee, ginseng coffee flavoured drinks
- Instant coffees
- Coffee pods for hot drinks
- Tea, camomile, herbal teas (liquid, soluble and prepared in powder form)
- Tea, decaffeinated tea, herbal teas (loose, filter, sachet) consisting of herbs, fruits and flavourings with other ingredients (e.g. chocolate, meringue, caramel)

FORBIDDEN FOODS

- Soluble coffee, coffee substitutes, beverages and preparations made from prohibited cereals (e.g. barley) except those labelled gluten-free

ALLOWED FOODS

- ✓ Wine, sparkling wine even if sulphites are added
- ✓ Distillates (cognac, gin, grappa, rum, tequila, whisky, vodka) without added flavourings or other substances

UNSAFE FOODS (TO CHECK)

- Alcoholic beverages with added flavourings or other substances (e.g. liqueurs, spirits with added substances)
- Beers made from permitted cereals
- Cider

FORBIDDEN FOODS

- Beers made from barley and/or wheat malt, except those labelled gluten-free

ALLOWED FOODS

- ✓ Honey, sugar (white, cane, granulated)
- ✓ Jams, marmalades, fruit jellies and compotes, chestnut creams
- ✓ Raw liquorice root
- ✓ Maltodextrins and glucose syrups, including dextrose, including those derived from prohibited cereals
- ✓ Pure fructose, stevia (also in powder form without
- ✓ the addition of other ingredients)
- ✓ Syrups from: agave, maple, rice, etc...

UNSAFE FOODS (TO CHECK)

- Cocoa powder
- Candy, candied fruit, sweets, jellies, chewing gum
- Chocolate (with and without filling), spreads
- Cake decorations (pralines, sprinkles, food colouring)
- Sweeteners: sorbitol, maltitol, mannitol, isomalt, xylitol,
- erythritol, stevia-based, aspartame, saccharin, etc.
- Industrial or artisanal ice creams, semi-finished products for home-made ice creams/ice creams
- Marrons glacés
- Sugar paste, marzipan
- Fruit preparations
- Nougat, crunchy
- Icing sugar, flavoured

FORBIDDEN FOODS

- Chocolate with prohibited cereals
- Cakes, biscuits and pastries made with prohibited flours and/or unsuitable ingredients

10. FATS, SPICES, SEASONINGS AND OTHER

ALLOWED FOODS

- ✓ Butter, lactose-free butter, clarified butter, lard, cocoa butter
- ✓ Vegetable oils
- ✓ Wine vinegar (unflavoured) and apple vinegar
- ✓ Traditional Balsamic Vinegar DOP: of Modena, of Reggio Emilia; Balsamic Vinegar of Modena PGI
- ✓ Balsamic Condiments not DOP and not IGP if they are made only from cooked grape must, wine vinegar and sulphites
- ✓ Yeast (brewer's yeast): fresh, freeze-dried, dry
- ✓ Sodium bicarbonate, ammonia for sweets, cream of tartar as such
- ✓ Yeast extract
- ✓ Agar-Agar leaves
- ✓ Royal jelly, pollen
- ✓ Tomato puree, peeled tomatoes, tomato pulp and tomato concentrate not mixed with other ingredients except ascorbic acid (E300 or vitamin C) and citric acid (E330), tomato juice, sugar, salt
- ✓ Pepper, salt, saffron
- ✓ Spices and aromatic herbs as they are, even if salt is added
- ✓ Curry consisting solely of spices and herbs
- ✓ Lemon juice with no other added ingredients (excluding metabisulphites and essential oil)
- ✓ Seeds (hemp, chia, sunflower, linseed, sesame, pumpkin, etc.) in their unaltered state or roasted and salted without the addition of other ingredients

UNSAFE FOODS (TO CHECK)

- Flavoured vinegar, non-DOP and non-IGP balsamic condiments with added ingredients other than cooked grape must, wine vinegar and sulphites
- Glaze, cream based on vinegar and/or other ingredients
- Béchamel with flours from permitted cereals
- Light butter, margarine and light margarine
- Condiments with undefined composition
- Ready-made sauces (ragout, pesto, etc.)
- Seed mixes
- Sauces (mayonnaise, mustard, ketchup, etc.), pâté, anchovy paste
- Mustard
- Stock cubes, stock preparations, extracts (meat and vegetable)
- Flavouring agents consisting not only of spices and salt but also of other
- other ingredients (flavourings, starches, etc.)
- Agar-Agar powder, in bars
- Guar gum, xanthan gum
- Flavourings - vanillin
- Fish glue, food gelatine, thickeners
- Chemical yeast (leavening agents)
- Fresh liquid brewer's yeast
- Sourdough from permitted cereals
- Soya lecithin
- Miso, tamari, soy sauce
- Curry with other ingredients
- Tofu
- Food supplements

FORBIDDEN FOODS

- Sourdough
- Seitan
- Béchamel with flours from prohibited cereals
