# Supplementary material for: Novel probiotic preparation with in vivo gluten-degrading activity and potential modulatory effects on the gut microbiota
Source: Microbiol Spectr. 2024 Jun 11;12(7):e03524-23. doi: 10.1128/spectrum.03524-23 (PMC11218521; doi:10.1128/spectrum.03524-23)
Supplement: Table S1 — Differentially abundant taxa. [file spectrum.03524-23-s0003.docx]

| Table S1. Differentially abundant taxa between placebo and probiotic group as calculated using Lefse algorithm. | | | | |
| --- | --- | --- | --- | --- |
| **Genus** | **Logaritmic value of the class with the highest mean** | **class with the highest mean** | **logarithmic LDA** | **p-value** |
| Blautia | 5.170102417 | Placebo | 4.301472149 | 0.020709145 |
| Ruminococcus_torques_group | 4.228291903 | Placebo | 3.373957325 | 0.049171345 |
| Family_XIII_AD3011_group | 3.048507946 | Placebo | 2.376251906 | 0.041885997 |
| Clostridiuminnocuum_group | 2.526679831 | Placebo | 2.227245853 | 0.009552418 |
| Oscillibacter | 2.876024002 | Placebo | 2.081590251 | 0.006725258 |
| Flavonifractor | 2.64079907 | Placebo | 2.003762282 | 0.000366613 |
| Coprococcus | 4.216062662 | Probiotic | 3.72676634 | 0.000114214 |
| unspecific_Eubacterium_coprostanoligenes_group | 4.272578858 | Probiotic | 3.478129635 | 0.00432316 |
| Christensenellaceae_R-7_group | 4.192666538 | Probiotic | 3.35897058 | 0.01104749 |
| Lachnospiraceae_NK4A136_group | 3.894221716 | Probiotic | 3.295926493 | 0.006558151 |
| unspecific_ClostridiaUCG_014 | 4.032630941 | Probiotic | 3.27618853 | 0.002651179 |
| Butyrivibrio | 3.414995086 | Probiotic | 3.078459698 | 0.029316301 |
| CAG_56 | 3.446924614 | Probiotic | 2.834874971 | 0.002507659 |
| unspecific_UCG_011 | 3.565486086 | Probiotic | 2.802217113 | 0.00707787 |
| Akkermansia | 3.494048082 | Probiotic | 2.764805942 | 0.029413984 |
| Streptococcus | 3.673627291 | Probiotic | 2.723255297 | 0.021655277 |
| unspecific_EPR3968_O8a_Bc78 | 0.492726214 | Probiotic | 2.656963625 | 0.039148394 |
| NK4A214group | 3.182712612 | Probiotic | 2.624551739 | 0.02755755 |
| Eubacterium_xylanophilum_group | 3.169633738 | Probiotic | 2.525596288 | 0.006727447 |
| Lachnospiraceae_FCS020_group | 3.24858474 | Probiotic | 2.495308291 | 0.028771154 |
| unspecific_TK17 | 0.643683743 | Probiotic | 2.47060046 | 0.026337334 |
| unspecific_bacteriumGBS_1 | 0.649789407 | Probiotic | 2.359109981 | 0.001543926 |
| Cloacibacillus | 0.709729966 | Probiotic | 2.306291704 | 0.004746124 |
| Eubacterium_ruminantium_group | 3.14577853 | Probiotic | 2.298740692 | 0.02639312 |
| unspecific_Desulfitobacteriales | 1.214052105 | Probiotic | 2.290610379 | 0.048387836 |
| Lactococcus | 2.647804646 | Probiotic | 2.280317183 | 0.010148276 |
| Herbinix | 0.814097183 | Probiotic | 2.274281775 | 0.048528118 |
| Phocea | 0.847941658 | Probiotic | 2.269160517 | 0.044017245 |
| unspecific_Clostridiales | 0.968518625 | Probiotic | 2.253266512 | 0.014219611 |
| LachnospiraceaeUCG_007 | 1.054054568 | Probiotic | 2.239156114 | 0.000975727 |
| unspecific_Caloranaerobacter | 0.892641686 | Probiotic | 2.233142012 | 0.025321649 |
| unspecific_unidentified_rumen_bacterium_RFN82 | 1.010259141 | Probiotic | 2.212415276 | 0.039148394 |
| Fusobacterium | 0.764202666 | Probiotic | 2.201721311 | 0.023954981 |
| LachnospiraceaeUCG_009 | 1.267335675 | Probiotic | 2.180173956 | 0.048309255 |
| unspecific_Rhodocyclaceae | 1.073308588 | Probiotic | 2.17214144 | 0.009754982 |
| Methanosphaera | 1.608129203 | Probiotic | 2.114274675 | 0.044422141 |
| unspecific_Gastranaerophilales | 2.68621795 | Probiotic | 2.084434842 | 0.022674518 |
| unspecific_Caloramatoraceae | 1.113114177 | Probiotic | 2.073035685 | 0.00424757 |
| unspecific_Lactobacillaceae | 1.391070137 | Probiotic | 2.005381678 | 0.007408694 |
