# Supplementary material for: Novel probiotic preparation with in vivo gluten-degrading activity and potential modulatory effects on the gut microbiota
Source: Microbiol Spectr. 2024 Jun 11;12(7):e03524-23. doi: 10.1128/spectrum.03524-23 (PMC11218521; doi:10.1128/spectrum.03524-23)
Supplement: Table S2 — Volcano plot results T1. [file spectrum.03524-23-s0004.docx]

| Table S2. Volcano plot results T1 |  |  |  |  |
| --- | --- | --- | --- | --- |
| Compounds | FC | log2(FC) | p.ajusted | minusLOG10(p) |
| Copaene | 114.2 | 6.8354 | 5.82E-07 | 6.2348 |
| 1,7-Hexadecadiene | 18.188 | 4.1849 | 2.19E-06 | 5.6597 |
| alpha-Pinene | 123.52 | 6.9486 | 1.01E-05 | 4.994 |
| (E)-Tetradec-2-enal | 70.22 | 6.1338 | 1.91E-05 | 4.7194 |
| 2,5-Dihydroxybenzaldehyde, 2TM | 7.1871 | 2.8454 | 0.00013 | 3.887 |
| Tetradecanal | 13.048 | 3.7057 | 0.000407 | 3.3905 |
| trans-gamma-Bisabolene | 15.767 | 3.9789 | 0.000527 | 3.2784 |
| 2-Tetradecanone | 11.281 | 3.4958 | 0.000527 | 3.2784 |
| 2-Nonanone | 0.15167 | -2.721 | 0.000705 | 3.1519 |
| Hexanal | 0.097718 | -3.3552 | 0.00088 | 3.0555 |
| (2E,4E)-3,7-Dimethylocta-2,4-d | 2.3658 | 1.2423 | 0.00088 | 3.0555 |
| Caryophyllene | 6.9612 | 2.7993 | 0.001084 | 2.9648 |
| 1H-Pyrrole-2,5-dione, 3-ethyl- | 2.6057 | 1.3817 | 0.001192 | 2.9237 |
| Beta-Bisabolene | 94.162 | 6.5571 | 0.001238 | 2.9072 |
| Pentanoic acid, ethyl ester | 3.7261 | 1.8977 | 0.001544 | 2.8113 |
| Caparratriene | 2.2363 | 1.1611 | 0.002077 | 2.6826 |
| 3-Carene | 8.1112 | 3.0199 | 0.002689 | 2.5704 |
| 1,2-Benzenediol, 3,5-bis(1,1-d | 2.7004 | 1.4331 | 0.002689 | 2.5704 |
| Pentanoic acid, butyl ester | 10.04 | 3.3276 | 0.002858 | 2.544 |
| Tetradecane | 2.7808 | 1.4755 | 0.004039 | 2.3938 |
| Methyl valerate | 7.5631 | 2.919 | 0.004616 | 2.3357 |
| Propanal, 2-methyl- | 2.4164 | 1.2729 | 0.004745 | 2.3238 |
| Pentanoic acid, pentyl ester | 95.528 | 6.5778 | 0.005929 | 2.227 |
| Cadina-1(10),4-diene | 25.994 | 4.7001 | 0.005929 | 2.227 |
| Butanoic acid, 2-methyl-, ethy | 6.3958 | 2.6771 | 0.005929 | 2.227 |
| 2-Pentanone | 2.2596 | 1.1761 | 0.005929 | 2.227 |
| Pentanoic acid, 4-methyl- | 4.3998 | 2.1374 | 0.007489 | 2.1256 |
| 2-Pentadecanone | 2.0346 | 1.0248 | 0.010248 | 1.9894 |
| n-Decanoic acid | 3.0222 | 1.5956 | 0.011824 | 1.9272 |
| 1-Hexadecanol | 5.3892 | 2.4301 | 0.013536 | 1.8685 |
| 2-Undecanone | 0.45972 | -1.1212 | 0.018477 | 1.7334 |
| alpha-Phellandrene | 60.333 | 5.9149 | 0.019262 | 1.7153 |
| beta-Phellandrene | 54.361 | 5.7645 | 0.019262 | 1.7153 |
| gamma-Terpinene | 35.417 | 5.1464 | 0.019262 | 1.7153 |
| 2-Hexadecene, 3,7,11,15-tetram | 8.036 | 3.0065 | 0.019262 | 1.7153 |
| Acetyl valeryl | 0.43137 | -1.213 | 0.019985 | 1.6993 |
| 2-Octenal, (E)- | 0.060101 | -4.0565 | 0.020584 | 1.6865 |
| 1-Butanol, 3-methyl- | 0.4703 | -1.0883 | 0.03166 | 1.4995 |
| Citral | 0.093248 | -3.4228 | 0.041048 | 1.3867 |
