# Supplementary material for: Novel probiotic preparation with in vivo gluten-degrading activity and potential modulatory effects on the gut microbiota
Source: Microbiol Spectr. 2024 Jun 11;12(7):e03524-23. doi: 10.1128/spectrum.03524-23 (PMC11218521; doi:10.1128/spectrum.03524-23)
Supplement: Table S3 — Volcano plot results T5. [file spectrum.03524-23-s0005.docx]

| Table S3. Volcano plot results T5 |  |  |  |  |
| --- | --- | --- | --- | --- |
| Compounds | FC | log2(FC) | p.ajusted | minusLOG10(p) |
| Butanoic acid, 3-methylbutyl e | 274.43 | 8.1003 | 1.45E-06 | 5.84 |
| Methyl valerate | 237 | 7.8887 | 2.85E-06 | 5.5452 |
| Cadina-1(10),4-diene | 64.577 | 6.0129 | 2.85E-06 | 5.5452 |
| Pentanoic acid, butyl ester | 104.11 | 6.7019 | 6.58E-06 | 5.1818 |
| Propanoic acid, ethyl este | 14.548 | 3.8627 | 5.36E-05 | 4.2707 |
| 1-Pentadecene | 0.033677 | -4.8921 | 6.17E-05 | 4.2096 |
| Butanoic acid, propyl ester | 20.424 | 4.3522 | 6.17E-05 | 4.2096 |
| Benzeneacetic acid, ethyl este | 6.2884 | 2.6527 | 6.17E-05 | 4.2096 |
| 2-Tetradecanone | 32.49 | 5.0219 | 9.06E-05 | 4.0431 |
| Pentanoic acid, 4-methyl- | 18.218 | 4.1873 | 9.06E-05 | 4.0431 |
| Pentanoic acid, ethyl ester | 4.7847 | 2.2584 | 9.06E-05 | 4.0431 |
| Levomenthol | 13.039 | 3.7047 | 0.00015636 | 3.8059 |
| 1-Butanol, 3-methyl- | 0.16656 | -2.5858 | 0.0001645 | 3.7838 |
| Copaene | 7.4412 | 2.8955 | 0.00027543 | 3.56 |
| 1-Tetracosene | 0.23249 | -2.1047 | 0.00095186 | 3.0214 |
| Benzenepropanoic acid, ethyl e | 4.0793 | 2.0283 | 0.00095186 | 3.0214 |
| Pentanoic acid | 2.5294 | 1.3388 | 0.00095186 | 3.0214 |
| (-)-beta-Pinene | 20.932 | 4.3876 | 0.0011115 | 2.9541 |
| Butanoic acid, ethyl ester | 4.9881 | 2.3185 | 0.0011115 | 2.9541 |
| 2,5-Dihydroxybenzaldehyde, 2TM | 0.39945 | -1.3239 | 0.0013636 | 2.8653 |
| Pentadecane | 0.37236 | -1.4252 | 0.002233 | 2.6511 |
| 3-Carene | 5.3262 | 2.4131 | 0.0024124 | 2.6175 |
| Caryophyllene | 3.1891 | 1.6732 | 0.0024124 | 2.6175 |
| Butanoic acid, pentyl ester | 455.11 | 8.8301 | 0.0027843 | 2.5553 |
| Pentanoic acid, pentyl ester | 366 | 8.5157 | 0.0027843 | 2.5553 |
| Furan, 3-(4-methyl-3-pentenyl) | 134.57 | 7.0722 | 0.0027843 | 2.5553 |
| Butanoic acid, butyl ester | 108.38 | 6.76 | 0.0027843 | 2.5553 |
| Citral | 95.214 | 6.5731 | 0.0027843 | 2.5553 |
| Butanoic acid, 2-methyl-, ethy | 37.027 | 5.2105 | 0.0027843 | 2.5553 |
| Pentanoic acid, propyl ester | 36.321 | 5.1827 | 0.0027843 | 2.5553 |
| Butanoic acid, 2-methyl-, prop | 14.891 | 3.8964 | 0.0027843 | 2.5553 |
| 1-Butanol, 2-methyl- | 0.35188 | -1.5068 | 0.0027843 | 2.5553 |
| 2-Nonenal, (E)- | 0.1917 | -2.3831 | 0.0031418 | 2.5028 |
| Tetradecane | 4.019 | 2.0068 | 0.003383 | 2.4707 |
| n-Decanoic acid | 25.429 | 4.6684 | 0.022166 | 1.6543 |
| 2-Pentadecanone | 2.6998 | 1.4329 | 0.022166 | 1.6543 |
| (E)-Tetradec-2-enal | 98.286 | 6.6189 | 0.030029 | 1.5225 |
| 2-Hexadecanone | 35.647 | 5.1557 | 0.030029 | 1.5225 |
| Heptanoic acid, ethyl ester | 27.08 | 4.7592 | 0.030029 | 1.5225 |
| 3-Pentanol | 0.0088848 | -6.8144 | 0.034636 | 1.4605 |
| Dimethyl trisulfide | 0.043816 | -4.5124 | 0.034636 | 1.4605 |
| Acetophenone, 4'-amino- | 0.064053 | -3.9646 | 0.034636 | 1.4605 |
| 2-Hexadecene, 3,7,11,15-tetram | 0.10974 | -3.1879 | 0.034636 | 1.4605 |
| gamma-Terpinene | 2.2692 | 1.1822 | 0.034636 | 1.4605 |
| D-Limonene | 2.3016 | 1.2026 | 0.041427 | 1.3827 |
| Methyl Isobutyl Ketone | 2.1136 | 1.0797 | 0.0447 | 1.3497 |
