# Supplementary material for: Novel probiotic preparation with in vivo gluten-degrading activity and potential modulatory effects on the gut microbiota
Source: Microbiol Spectr. 2024 Jun 11;12(7):e03524-23. doi: 10.1128/spectrum.03524-23 (PMC11218521; doi:10.1128/spectrum.03524-23)
Supplement: Table S4 — Volcano plot results T6. [file spectrum.03524-23-s0006.docx]

| Table S4. Volcano plot results T6 |  |  |  |  |
| --- | --- | --- | --- | --- |
| Compounds | FC | log2(FC) | p.ajusted | minusLOG10(p) |
| 2,5-Dihydroxybenzaldehyde, 2TM | 35.214 | 5.1381 | 8.55E-06 | 5.0681 |
| alpha-Pinene | 19.049 | 4.2517 | 0.000357 | 3.4476 |
| Butanoic acid, butyl ester | 0.076883 | -3.7012 | 0.000357 | 3.4476 |
| Methyl valerate | 0.11508 | -3.1192 | 0.000414 | 3.3831 |
| 2-Nonenal, (E)- | 21.262 | 4.4102 | 0.000651 | 3.1862 |
| beta-Myrcene | 16.035 | 4.0032 | 0.000651 | 3.1862 |
| 1,2-Benzenediol, 3,5-bis(1,1-d | 13.333 | 3.7369 | 0.000651 | 3.1862 |
| 1-Butanol, 3-methyl- | 10.055 | 3.3298 | 0.000651 | 3.1862 |
| Methyl Isobutyl Ketone | 9.8951 | 3.3067 | 0.000651 | 3.1862 |
| Pentanoic acid, butyl ester | 0.10232 | -3.2888 | 0.000651 | 3.1862 |
| Propanoic acid, ethyl este | 0.17289 | -2.532 | 0.000651 | 3.1862 |
| Benzenepropanoic acid, ethyl ester | 0.23913 | -2.0641 | 0.000651 | 3.1862 |
| Propanal, 2-methyl- | 4.1766 | 2.0623 | 0.000651 | 3.1862 |
| Hexanoic acid, ethyl ester | 0.24603 | -2.0231 | 0.000835 | 3.0782 |
| Valencene | 0.21585 | -2.2119 | 0.000868 | 3.0616 |
| (E)-Tetradec-2-enal | 6.3672 | 2.6706 | 0.001053 | 2.9774 |
| Butanoic acid, propyl ester | 0.21445 | -2.2213 | 0.001053 | 2.9774 |
| 2-Pentadecanone | 2.8006 | 1.4857 | 0.001053 | 2.9774 |
| Copaene | 5.1143 | 2.3545 | 0.001387 | 2.8578 |
| 2-Tetradecanone | 6.259 | 2.6459 | 0.001598 | 2.7965 |
| o-Cymene | 8.4903 | 3.0858 | 0.002258 | 2.6464 |
| Butanoic acid, ethyl ester | 0.35497 | -1.4942 | 0.002724 | 2.5649 |
| 2-Hexadecene, 3,7,11,15-tetram | 14.832 | 3.8906 | 0.003874 | 2.4119 |
| Pentanoic acid, ethyl ester | 0.30719 | -1.7028 | 0.005507 | 2.2591 |
| Benzeneacetic acid, ethyl este | 0.29122 | -1.7798 | 0.008298 | 2.081 |
| 1-Hexanol | 0.41135 | -1.2816 | 0.009347 | 2.0293 |
| Hexadecanal | 2.0963 | 1.0679 | 0.010473 | 1.9799 |
| alpha-Phellandrene | 231.61 | 7.8555 | 0.01154 | 1.9378 |
| Beta-Bisabolene | 2.234 | 1.1596 | 0.01154 | 1.9378 |
| gamma-Undecalactone | 0.016686 | -5.9052 | 0.029164 | 1.5352 |
| Pentanoic acid, propyl ester | 0.046178 | -4.4366 | 0.029164 | 1.5352 |
| Anethole | 0.10385 | -3.2674 | 0.029164 | 1.5352 |
| Butanoic acid, pentyl ester | 34.893 | 5.1249 | 0.030744 | 1.5122 |
| 2-Octenal, (E)- | 26.143 | 4.7083 | 0.030744 | 1.5122 |
| Decane | 20.25 | 4.3399 | 0.030744 | 1.5122 |
| Phenol, 2-methyl-5-(1-methylet | 12.138 | 3.6014 | 0.030744 | 1.5122 |
| 2-Hexadecanone | 3.6032 | 1.8493 | 0.030744 | 1.5122 |
