# Supplementary material for: Novel probiotic preparation with in vivo gluten-degrading activity and potential modulatory effects on the gut microbiota
Source: Microbiol Spectr. 2024 Jun 11;12(7):e03524-23. doi: 10.1128/spectrum.03524-23 (PMC11218521; doi:10.1128/spectrum.03524-23)
Supplement: Table S5 — Fecal VOC probiotic group. [file spectrum.03524-23-s0007.docx]

Table S5. Faecal VOC probiotic group. Concentration (ppm) of statistically significant faecal VOC detected by assessing the probiotic group after 10 days of GFD (T1), 20 days of 10 g/day gluten intake (T5), of which 10 last days were the wash-out (T6).

|  | Probiotic_T1_ | Probiotic_T5_ | Probiotic_T6_ | T1 vs. T5 | T1 vs. T6 | T5 vs. T6 |
| --- | --- | --- | --- | --- | --- | --- |
| Compounds | ppm (sd) | ppm (sd) | ppm (sd) | Adjusted P Value* | | |
| (E)-Tetradec-2-enal | 0.005(0.004) | n.d. | 0.058(0.025) | ns | ns | 0.0012 |
| Indole, 3-methyl | 26.997(2.773) | 29.559(3.571) | 18.682(2.763) | ns | 0.0498 | 0.0372 |
| 1-Butanol | 0.092(0.011) | 0.026(0.004) | 0.102(0.021) | ns | ns | 0.0372 |
| 1-Butanol, 2-methyl- | 0.2(0.023) | 0.174(0.019) | 0.01(0.004) | ns | <0.0001 | <0.0001 |
| 1-Butanol, 3-methyl- | 0.4(0.06) | 0.346(0.036) | 0.017(0.011) | ns | <0.0001 | <0.0001 |
| 1-Hexadecanol | 0.063(0.024) | 0.156(0.02) | 0.269(0.077) | 0.0017 | <0.0001 | ns |
| 1-Hexanol | 0.132(0.013) | 0.077(0.012) | 0.237(0.031) | 0.018 | ns | <0.0001 |
| 1-Pentadecene | 0.085(0.033) | 0.905(0.125) | 0.093(0.051) | <0.0001 | ns | <0.0001 |
| 1-Tetracosene | 0.002(0.001) | 0.113(0.027) | 0.013(0.01) | <0.0001 | ns | <0.0001 |
| 1,7-Hexadecadiene | n.d. | 0.03(0.014) | 0.427(0.137) | ns | <0.0001 | <0.0001 |
| 1H-Pyrrole-2,5-dione, 3-ethyl- | 0.265(0.026) | 0.405(0.043) | 0.558(0.115) | ns | 0.0111 | ns |
| 2-Hexadecene, 3,7,11,15-tetram | n.d. | 0.063(0.025) | n.d. | 0.0001 | ns | 0.0001 |
| 2-Methoxy-4-vinylphenol | 0.017(0.006) | 0.043(0.021) | 0.124(0.02) | ns | <0.0001 | <0.0001 |
| 2-Nonenal, (E)- | 0.017(0.008) | 0.269(0.084) | 0.01(0.002) | <0.0001 | ns | <0.0001 |
| 2-Octenal, (E)- | 0.033(0.014) | 0.001(0.001) | n.d. | <0.0001 | <0.0001 | ns |
| 2-Pentanone | 0.032(0.011) | 0.056(0.008) | 0.157(0.049) | ns | ns | 0.0015 |
| 2-Undecanone | 0.345(0.04) | 0.43(0.052) | 0.214(0.041) | ns | 0.0239 | 0.0011 |
| Dihydroxybenzaldehyde | 0.009(0.005) | 0.038(0.005) | n.d. | <0.0001 | ns | <0.0001 |
| 3-Carene | 0.051(0.018) | 0.082(0.035) | 0.309(0.041) | ns | <0.0001 | <0.0001 |
| 5-Hepten-2-one, 6-methyl- | 2.068(0.218) | 3.451(0.348) | 4.054(0.714) | 0.0058 | 0.0019 | ns |
| 5,9-Undecadien-2-one, 6,10-dimethyl | 0.309(0.037) | 0.154(0.021) | 0.155(0.027) | 0.0012 | 0.0005 | ns |
| Acetyl valeryl | 0.152(0.051) | 0.026(0.018) | 0.039(0.016) | <0.0001 | 0.0002 | ns |
| alpha-Pinene | n.d. | 0.121(0.028) | 0.013(0.005) | <0.0001 | ns | <0.0001 |
| Benzeneacetic acid, ethyl ester | 0.039(0.012) | 0.019(0.012) | 0.213(0.062) | ns | <0.0001 | <0.0001 |
| Benzenepropanoic acid, ethyl ester | 0.039(0.013) | 0.041(0.019) | 0.335(0.071) | ns | <0.0001 | <0.0001 |
| Beta-Bisabolene | 0.003(0.002) | 0.993(0.36) | 0.166(0.032) | <0.0001 | <0.0001 | ns |
| beta-Myrcene | n.d. | 0.123(0.025) | 0.011(0.008) | <0.0001 | ns | <0.0001 |
| Butanoic acid, 2-methyl-, ethyl ester | n.d. | n.d. | 0.023(0.01) | ns | <0.0001 | <0.0001 |
| Butanoic acid, butyl ester | 0.037(0.022) | n.d. | 0.181(0.026) | ns | <0.0001 | <0.0001 |
| Butanoic acid, ethyl ester | 0.267(0.066) | 0.094(0.033) | 0.653(0.088) | 0.0323 | 0.0005 | <0.0001 |
| Butanoic acid, propyl ester | 0.012(0.005) | 0.004(0.003) | 0.099(0.028) | ns | <0.0001 | <0.0001 |
| Cadina-1(10),4-diene | n.d. | n.d. | 0.062(0.025) | ns | <0.0001 | <0.0001 |
| Caryophyllene | 0.561(0.069) | 1.12(0.169) | 3.489(0.817) | 0.0343 | <0.0001 | 0.0015 |
| D-Limonene | 0.616(0.14) | 0.637(0.059) | 2.192(0.462) | ns | <0.0001 | 0.001 |
| Furan, 3-(4-methyl-3-pentenyl) | 0.002(0.001) | n.d. | 0.01(0.004) | ns | ns | 0.0015 |
| Heptanoic acid | 0.613(0.07) | 0.794(0.095) | 3.105(0.551) | ns | <0.0001 | 0.001 |
| Hexanal | 0.96(0.304) | 0.175(0.03) | 0.14(0.034) | 0.0008 | <0.0001 | ns |
| Hexanoic acid | 1.865(0.216) | 2.441(0.286) | 4.182(0.73) | ns | 0.001 | ns |
| Hexanoic acid, ethyl ester | 0.213(0.132) | 0.006(0.002) | 0.773(0.125) | ns | <0.0001 | <0.0001 |
| Levomenthol | 0.35(0.047) | 0.032(0.022) | 0.123(0.052) | <0.0001 | <0.0001 | ns |
| Methyl Isobutyl Ketone | 0.035(0.005) | 0.024(0.006) | 0.005(0.002) | ns | <0.0001 | 0.0002 |
| Methyl valerate | 0.007(0.004) | n.d. | 0.15(0.039) | ns | <0.0001 | <0.0001 |
| n-Decanoic acid | 0.026(0.011) | 0.011(0.004) | 0.164(0.027) | ns | <0.0001 | <0.0001 |
| Nonanal | 0.048(0.012) | 0.107(0.014) | 0.177(0.03) | 0.0081 | <0.0001 | ns |
| Pentadecanal | 0.116(0.014) | 0.04(0.022) | 0.179(0.039) | <0.0001 | ns | <0.0001 |
| Pentadecane | 0.337(0.033) | 0.46(0.066) | 0.159(0.024) | ns | 0.0005 | <0.0001 |
| Pentanoic acid | 2.864(0.504) | 5.184(0.869) | 10.77(2.369) | 0.0456 | <0.0001 | ns |
| Pentanoic acid, 4-methyl- | 0.047(0.017) | 0.023(0.008) | 0.259(0.046) | ns | <0.0001 | <0.0001 |
| Pentanoic acid, butyl ester | 0.005(0.003) | 0.001(0.001) | 0.147(0.027) | ns | <0.0001 | <0.0001 |
| Pentanoic acid, ethyl ester | 0.077(0.038) | 0.175(0.032) | 0.803(0.198) | 0.0104 | <0.0001 | 0.0005 |
| Phenylethyl alcohol | 0.585(0.063) | 0.934(0.102) | 0.996(0.154) | 0.0361 | 0.0456 | ns |
| Propanal, 2-methyl- | 0.181(0.069) | 0.661(0.074) | 0.179(0.076) | <0.0001 | ns | <0.0001 |
| Propanoic acid, ethyl ester | 0.005(0.003) | 0.006(0.004) | 0.065(0.012) | <0.0001 | ns | <0.0001 |
| Tetradecanal | 0.009(0.006) | 0.323(0.044) | 0.527(0.086) | <0.0001 | <0.0001 | ns |
| Valencene | 0.01(0.006) | 0.113(0.075) | 0.688(0.094) | ns | <0.0001 | <0.0001 |

nd, not detected; ns, not significative

*Kruskal-Wallis test corrected by Dunn’s test
