# Supplementary material for: Novel probiotic preparation with in vivo gluten-degrading activity and potential modulatory effects on the gut microbiota
Source: Microbiol Spectr. 2024 Jun 11;12(7):e03524-23. doi: 10.1128/spectrum.03524-23 (PMC11218521; doi:10.1128/spectrum.03524-23)
Supplement: Table S6 — Concentration (ppm) of statistically significant fecal VOC. [file spectrum.03524-23-s0008.docx]

**Table S6.** Concentration (ppm) of statistically significant faecal VOC detected by assessing the placebo group after 10 days of GFD (T1), 20 days of 10 g/day gluten intake (T5), of which 10 last days were the wash-out (T6).

|  | **Placebo_T1_** | **Placebo_T5_** | **Placebo_T6_** | **T1 vs. T5** | **T1 vs. T6** | **T5 vs. T6** |
| --- | --- | --- | --- | --- | --- | --- |
| Compounds | ppm | ppm | ppm | Adjusted P Value* | | |
| Propanoic acid, ethyl ester | 0.007(0.004) | 0.017(0.011) | 0.011(0.009) | <0.0001 | ns | 0.0001 |
| beta-Myrcene | 0.004(0.002) | 0.018(0.173) | 0.173(0.035) | 0.0178 | <0.0001 | ns |
| Butanoic acid, propyl ester | 0.007(0.004) | 0.021(0.021) | 0.021(0.015) | 0.0002 | ns | 0.0015 |
| Pentanoic acid, ethyl ester | 0.298(0.053) | 0.174(0.245) | 0.245(0.064) | 0.0365 | ns | 0.0027 |

nd, not detected; ns, not significative

*Kruskal-Wallis test corrected by Dunn’s test
