# Supplementary material for: Novel probiotic preparation with in vivo gluten-degrading activity and potential modulatory effects on the gut microbiota
Source: Microbiol Spectr. 2024 Jun 11;12(7):e03524-23. doi: 10.1128/spectrum.03524-23 (PMC11218521; doi:10.1128/spectrum.03524-23)
Supplement: Table S7 — Mediterranean Diet Serving Score. [file spectrum.03524-23-s0009.docx]

| Table S7: Mediterranean Diet Serving Score (MDSS)^1^ | | |  |
| --- | --- | --- | --- |
| Food type | Recommendation* | Score |  |
| Fruit | 1–2 servings/main meal** | 3 |  |
| Vegetables | ≥2 servings/main meal** | 3 |  |
| Cereals^a^ | 1–2 servings/main meal** | 3 |  |
| Potatoes | ≤3 servings/week | 1 |  |
| Olive Oil^b^ | 1 serving/main meal** | 3 |  |
| Nuts | 1–2 servings/day | 2 |  |
| Dairy products^c^ | 2 servings/day | 2 |  |
| Legumes | ≥2 servings/week | 1 |  |
| Eggs | 2–4 servings/week | 1 |  |
| Fish | ≥2 servings/week | 1 |  |
| White meatd | 2 servings/week | 1 |  |
| Red meat^e^ | < 2 servings/week | 1 |  |
| Sweets^f^ | ≤2 servings/week | 1 |  |
| Fermented beverages^g^ | 1–2 glass/day | 1 |  |
| Total score |  | 24 |  |
|  |  |  |  |
| *According with the new Mediterranean Diet Pyramid (1). | |  |  |
| ** Main meals: breakfast, lunch and dinner. | |  |  |
| a Bread, breakfast cereals, rice and pasta. | |  |  |
| b Olive oil used on salads or bread or for frying | |  |  |
| c Milk, yoghurt, cheese, ice-cream | |  |  |
| d Poultry |  |  |  |
| e Pork, beef, or lamb |  |  |  |
| f Sugar, candies, pastries, sweetened fruit juices, and soft drinks  g Wine and beer. | | | |

1. Bach-Faig A, Berry E, Lairon D, Reguant J, Trichopoulou A, Dernini S, Medina FX, Battino M, Belahsen R, Miranda G, Serra-Majem L. 2011. Mediterranean diet foundation expert group, mediterranean diet pyramid today. Science and cultural updates. Public Health Nutr 14:2274–2284.
